# Peptide Amphiphiles Hitchhike on Endogenous Biomolecules for Enhanced Cancer Imaging and Therapy

**DOI:** 10.1002/adma.202509359

**Published:** 2025-10-04

**Authors:** Li Xiang, Morgan R. Stewart, Kailin Mooney, Mingchong Dai, Samuel Drennan, Samantha Holland, Arnaud Quentel, Sinan Sabuncu, Benjamin R. Kingston, Isabel J. Dengos, Karla Bonic, Florian Goncalves, Xin Yi, Michael I. Henderson, Srivathsan Ranganathan, Bruce P. Branchaud, Leslie L. Muldoon, Ramon F. Barajas Jr, Jared M. Fischer, Adem Yildirim

**Affiliations:** ^1^ CEDAR Knight Cancer Institute School of Medicine Oregon Health and Science University Portland Oregon 97201 USA; ^2^ Neuro‐Oncology Blood‐Brain Barrier Program Oregon Health and Science University Portland Oregon 97239 USA; ^3^ Department of Biomedical Engineering School of Medicine Oregon Health and Science University Portland Oregon 97239 USA; ^4^ Department of Neurology School of Medicine Oregon Health and Science University Portland Oregon 97239 USA; ^5^ Advanced Imaging Research Center Oregon Health and Science University Portland Oregon 97239 USA; ^6^ Department of Radiology School of Medicine Oregon Health and Science University Portland Oregon 97239 USA; ^7^ Knight Cancer Institute School of Medicine Oregon Health and Science University Portland Oregon 97201 USA; ^8^ Department of Molecular and Medical Genetics School of Medicine Oregon Health and Science University Portland Oregon 97239 USA; ^9^ Division of Oncological Sciences Knight Cancer Institute School of Medicine Oregon Health and Science University Portland Oregon 97201 USA

**Keywords:** cancer nanomedicine, drug delivery, fluorescence imaging, lipoproteins, nano‐bio interactions, peptide amphiphiles, self‐assembly

## Abstract

The interactions of nanomaterials with biomolecules in vivo determine their biological fate. Here, it is shown that self‐assembled peptide amphiphile (PA) nanostructures can dynamically interact with endogenous biomolecules and take advantage of naturally occurring processes to target a broad range of solid tumors. In circulation, self‐assembled PA nanostructures disassemble and reassemble mainly with lipoproteins, which prolongs blood circulation and dramatically improves tumor accumulation and retention. Mechanistic studies suggested that PAs internalize into cancer cells by assembling with their cell membranes and independently of specific receptors. By exploiting these interactions, a PA developed in this study (namely Self‐Assembly ‐ Glutamic acid, SA‐E) demonstrates specific accumulation in various xenograft, syngeneic, patient‐derived xenograft, or transgenic rodent models. In addition, SA‐E enabled the effective delivery of highly potent chemotherapy to different syngeneic and xenografted tumors with reduced side effects. With its simple and modular design and universal tumor accumulation mechanism, SA‐E represents a promising platform for broad applications in cancer imaging and therapy.

## Graphical abstract

Weakly assembled peptide amphiphile nanostructures disassemble in circulation and reassemble with blood biomolecules, mainly lipoproteins. Binding to blood biomolecules prolongs blood circulation and improves accumulation in solid tumors. In the tumor microenvironment, peptide amphiphiles assemble with cholesterol‐rich domains of cell membranes, allowing them to selectively internalize in cancer cells and effectively deliver chemotherapies conjugated to them.

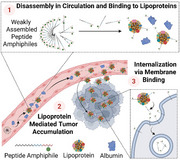

## 1 Introduction

Peptide amphiphiles (PAs) spontaneously self‐assemble into nanostructures in aqueous solutions through hydrophobic and electrostatic interactions and hydrogen bonding.^[^ ^1^, ^2^, ^3^, ^4^ ^]^ By exploiting the rich chemistry of amino acid side chains and abundant peptide modifications at both the side chains and terminal ends, PA nanostructures with various morphologies have been developed.^[^ ^5^, ^6^, ^7^ ^]^ PA nanostructures are naturally biodegradable and biocompatible with low immunogenicity, making them promising delivery platforms for biomedical applications.^[^ ^8^, ^9^, ^10^, ^11^ ^]^ Accordingly, PAs have been applied for various applications, from drug delivery to molecular imaging and tissue engineering.^[^ ^6^, ^12^, ^13^ ^]^ Like any other self‐assembled structure, the physically interacting PA monomers in their nanostructures can dynamically interact with biological structures and molecules such as cell membranes and proteins in vivo.^[^ ^14^, ^15^ ^]^ For instance, some recent studies showed that weakly assembled PA nanostructures can interact more effectively with cell membranes, affecting cell viability, and intracellular signaling pathways.^[^ ^16^, ^17^, ^18^, ^19^, ^20^ ^]^ Nevertheless, the primary focus in the field has been developing PA assemblies with good structural stability in biological environments to allow them to remain intact in vivo throughout their intended duration of application, and studies on their dynamic interactions with biological systems have been limited.^[^ ^15^, ^16^, ^17^, ^18^, ^19^, ^20^, ^21^, ^22^, ^23^ ^]^ In addition, studies in the last decades have demonstrated the importance of nano‐bio interactions on the biological fate of nanomaterials in vivo.^[^ ^24^, ^25^, ^26^ ^]^ It is now well known that the composition of the proteins that nanomaterials dynamically interact with (i.e., protein or biomolecular corona) determines their pharmacokinetics, biodistribution, and molecular targeting, which can be controlled through nanomaterial design (i.e., nanoparticle shape, surface charge, or stiffness).^[^ ^24^, ^25^, ^26^ ^]^ For example, adsorption of complement proteins on nanoparticle surfaces can result in their rapid clearance by the reticuloendothelial system.^[^ ^27^, ^28^ ^]^ On the contrary, interaction with proteins such as albumin or Apolipoprotein E can improve blood circulation and enable their receptor‐mediated (e.g., low‐density lipoprotein receptor, LDL‐R) transcytosis and accumulation in cancer cells.^[^ ^29^, ^30^, ^31^ ^]^ Despite the well‐documented importance of nano‐bio interactions for solid nanomaterials, these interactions have mostly remained unexplored for self‐assembled nanomaterials such as PA nanostructures.

In this work, we explored how PAs interact with endogenous biomolecules and cells and how these interactions affect their cancer targeting ability and biodistribution using various in vitro and in vivo models. Our studies revealed a mechanism allowing PAs to target and enter cancer cells independent of cell state or surface markers. Specifically, we found that weakly assembled PAs can assemble with blood biomolecules and cell membranes upon in vivo administration, allowing them to exploit the increased lipid metabolism of cancer cells to specifically accumulate in solid tumors. For these studies, we designed PAs that can self‐assemble into micelles with spherical or rod‐shaped morphologies, giving them different stabilities in blood plasma. Through molecular dynamic simulations and in vitro experiments, we found that intramolecular interactions in nanostructures of PAs with spherical morphology (namely SA‐E) are weaker than rod‐shaped ones (namely Self‐Assembly‐Lysine, SA‐K). Upon incubation with blood plasma, while more strongly interacting SA‐K micelles remained largely intact, SA‐E micelles quickly disassembled due to lower intermolecular interactions in these structures. Protein chromatography and mass spectrometry studies showed that SA‐E selectively binds to lipoproteins (LPs) in the blood, mainly high‐density lipoprotein (HDL), which are endogenous nanoparticles with sizes ∼5‐15 nm and composed of lipids, cholesterol, and amphiphilic proteins (i.e., apolipoproteins).^[^ ^32^ ^]^ HDL has a long blood circulation half‐life, intrinsic biocompatibility (i.e., non‐toxic and non‐immunogenic), and tumor accumulation as a result of increased lipid metabolism of cancer cells.^[^ ^32^, ^33^, ^34^, ^35^ ^]^ In addition, our studies indicated that PAs internalize into cells as monomers through membrane binding, not as intact micelles. Dynamic interactions of SA‐E with these endogenous biomolecules prolonged its blood circulation and enabled strong accumulation in a broad range of solid tumors compared to more stable SA‐K nanostructures. We also found that SA‐E was enriched in cancer cells in the tumor microenvironment. Biodistribution studies showed that SA‐E was mostly cleared from normal tissue in 2 days but retained in the tumor for more than 2 weeks. The substantial tumor accumulation and retention of SA‐E enabled the detection of millimeter‐sized early breast tumors and colon adenomas in mice and intracerebral glioma tumors in rats with tumor‐to‐healthy brain tissue ratios of ≈16. Finally, we conjugated SA‐E with a highly potent and toxic chemotherapeutic agent, Monomethyl auristatin E (MMAE), and demonstrated its strong antitumor efficacy in breast, glioma, and colon cancer models in mice with reduced side effects compared to the free drug.

## 2 Results and Discussion

### 2.1 Stability and Interactions of PA Nanostructures in Blood Plasma

**Figure 1:**
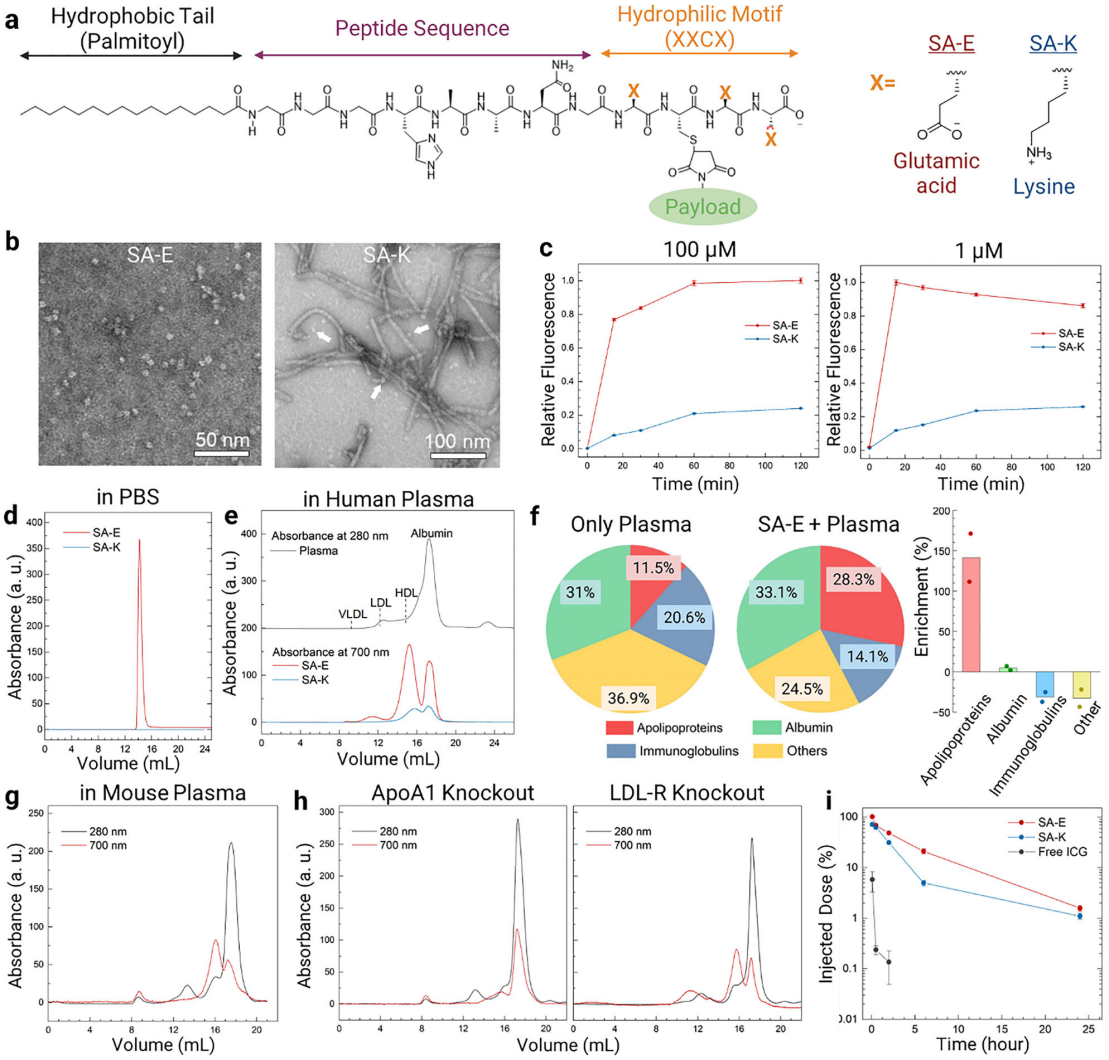
Spherical PA micelles disassemble in plasma and in situ reassemble with plasma biomolecules for prolonged blood circulation. a) Schematic showing the overall molecular structure of the PAs used in this study. b) TEM images of PA nanostructures. White arrows highlight the spherical micelles in SA‐K sample. Samples were negatively stained using uranyl acetate. c) Fluorescence intensity of ICG labeled PAs in 10% human plasma over 2 h. FPLC traces of PAs in PBS d) or 10% human plasma e). Absorbance at 280 nm was used to detect LPs and albumin, and 700 nm was used to detect SA‐E. f) Summary of protein classes identified in pulled‐down proteins in 10% human plasma samples with or without SA‐E by mass spectrometry (left panel). Percent enrichment of proteins in the presence of SA‐E (right panel). FPLC traces of plasma samples collected from wild type g) or ApoA1‐/‐ or LDL‐R‐/‐ h) mice injected with SA‐E (50 nmol) 1 hour before blood collection. i) Blood circulation of intravenously injected ICG labeled PAs or free ICG (50 nmol) in wild type mice. Data in (c, i) are presented as mean ± standard error of the mean (SEM). Bars in (f) are the mean of the data. Studies were run in triplicates in (c, i) and duplicates in (e).

The overall design of the PAs used in this study is shown in **Figure** **1**a. Both PAs share a common motif to promote self‐assembly through hydrophobic interactions and hydrogen bonding: GGGHAANG with a palmitic acid modification on the N‐terminal.^[^ ^36^, ^37^ ^]^ While keeping this motif the same, we tuned the hydrophilic part of the PAs to change the morphology of self‐assembled PA nanostructures.^[^ ^38^ ^]^ SA‐E and SA‐K contain three glutamic acids (E) or lysines (K) in their hydrophilic part, respectively. In addition, a cysteine residue was added for dye or drug conjugation through maleimide‐thiol coupling. PAs were conjugated with a near‐infrared (NIR) fluorescent dye (indocyanine green; ICG) to track the PA nanostructures in vivo and investigate their stability in biological solutions. PA‐ICG conjugates were purified using high‐performance liquid chromatography (HPLC) and characterized using liquid chromatography‐mass spectrometry (LC‐MS) (Figures S1 and S2, Supporting Information).

The morphology of self‐assembled nanostructures of ICG conjugated PAs was investigated using transmission electron microscopy (TEM) (Figure 1b). SA‐E demonstrated a spherical micelle morphology with sizes around 5–10 nm, and SA‐K formed mostly rod‐shaped micelles with diameters around ≈10 nm and lengths reaching several hundred nm. Some small spherical micelles were also observed in the SA‐K sample (white arrows in Figure 1b; Figure S3, Supporting Information). To evaluate the impact of ICG labeling on the morphology of PA micelles, TEM images of unlabeled PAs were also taken (Figure S4, Supporting Information), which showed a similar morphology to that of ICG‐conjugated PAs.

To understand the stability of self‐assembled PA nanostructures in biologically complex solutions, we initially utilized human plasma (Figure 1c). Before moving to these studies, we first confirmed that SA‐E and SA‐K have similar fluorescence properties at their assembled and disassembled states by disassembling their micelles using 5% Sodium dodecyl sulfate (SDS; Figure S5, Supporting Information). In the absence of SDS, the fluorescence of ICG was almost completely quenched in the micelles of both SA‐E and SA‐K due to the close packing of ICG molecules.^[^ ^39^ ^]^ The addition of SDS sharply increased the fluorescence of both PAs to a very similar degree, suggesting that PA micelles were disassembled and their monomers have similar fluorescence properties. We then incubated the PAs (1 or 100 µm) in 10% human plasma, and the fluorescence of ICG was measured at different time points. While the addition of plasma resulted in an increase in the fluorescence of both PAs, the increase in the SA‐E fluorescence was approximately three‐fold larger and more rapid than SA‐K at both concentrations, suggesting a better structural stability in plasma for SA‐K micelles compared to micelles of SA‐E. We also performed fluorescence polarization experiments as reported previously^[^ ^18^ ^]^ to further study the structural stability of PA micelles. For this study, we reduced the ICG concentration to 10% by mixing ICG conjugated PAs with their unconjugated counterparts to prevent complete fluorescence quenching of ICG. SA‐K showed a significantly higher anisotropy than SA‐E (Figure S6, Supporting Information), suggesting a more rigid structure for SA‐K micelles.^[^ ^18^ ^]^

To gain more insights on the stability of PAs, we also performed molecular dynamics (MD) simulations. For MD simulations, PAs were modeled into tube‐like structures, and they were observed throughout the simulation (≈0.83 µs). In accordance with the TEM observations, SA‐E formed spherical‐like clumps at the end of the simulation, but SA‐K mostly preserved the initial tube‐like orientation (Figure S7a,b, Supporting Information). To compare the structural stability of assemblies of SA‐E and SA‐K, we calculated the root mean square deviation (RMSD) of the PA structures and the number of intermolecular contacts between the heavy atoms of PAs throughout the simulation (Figure S7c,d, Supporting Information). Compared to SA‐E, SA‐K demonstrated a higher number of contacts with a smaller RMSD value, suggesting stronger intermolecular interactions in SA‐K micelles (See Supporting Information for further details). Overall, fluorescence experiments and MD simulations showed that rod‐shaped PA nanostructures of SA‐K are more stable in plasma than spherical ones of SA‐E.^[^ ^40^, ^41^ ^]^

Next, we sought to identify the blood components that PA nanostructures interact with. The most likely interactions of PAs in the blood are expected to be with albumin and LPs (e.g., very low‐density lipoprotein; VLDL, low‐density lipoprotein; LDL, or HDL), as they are highly abundant in blood and carry lipophilic molecules.^[^ ^32^, ^33^, ^34^, ^35^, ^42^, ^43^, ^44^, ^45^ ^]^ To study the interactions of PAs with these fractions of plasma, we performed fast protein liquid chromatography (FPLC) experiments using SA‐E and SA‐K in the absence or presence of pooled human plasma or purified LPs and albumin. Without plasma, SA‐E dispersed in PBS eluted from the column as a single narrow peak, as expected for self‐assembled micelles with a narrow size distribution of around 10 nm (Figure 1d). No peak was detected for SA‐K, suggesting that long rod‐shaped micelles of SA‐K were outside the size range of the FPLC column (≈5–5000 kDa) used in this study. Before moving to the studies with human plasma, we determined the elution times of albumin and LPs (VLDL, LDL, or HDL) in the FPLC using human serum albumin or LPs purified from human plasma (Figure S8a, Supporting Information). In addition, we performed FPLC using SA‐E mixed with purified LPs or albumin, which showed a lack of the SA‐E micelle peak observed in PBS. SA‐E eluted with the LPs or albumin (Figure S8b, Supporting Information), showing that SA‐E micelles completely disassembled and reassembled with these biomolecules. Similarly, in 10% human plasma (Figure 1e) there was no intact SA‐E micelle peak. Instead, SA‐E was found to be mainly bound to LPs (65.3% total, 59.8%, 5.3%, and 0.3% for HDL, LDL, and VLDL, respectively) and to a lower extent to albumin (34.7%). While the albumin peak in the FPLC trace at 280 nm was ≈20‐fold stronger than HDL, SA‐E bound to albumin (trace at 700 nm) was approximately two‐fold lower than HDL, suggesting a stronger affinity of SA‐E against HDL than albumin. The higher affinity of SA‐E toward LPs was also confirmed by performing FPLC of SA‐E incubated in a mixture of LPs and albumin, which showed almost entire binding to LPs (Figure S8c, Supporting Information). While a similar binding profile was observed for SA‐K in human plasma (Figure 1e) with higher binding to LPs (55% total, 50.7% and 4.3% for HDL and LDL, respectively) than albumin (45%), the area under the curve (AUC) of all peaks was ≈4.5 lower than SA‐E, which is in accordance with stability experiments above and further indicating that SA‐K micelles remain mostly structurally stable in plasma. In addition, we prepared a version of SA‐E without the n‐terminal lipid modification and conjugated with ICG (No‐SA; Figure S9, Supporting Information) to explore the impact of lipid modification on the assembly of PAs with plasma components. FPLC of No‐SA in 10% human plasma (Figure S10, Supporting Information) showed that it remained mostly unbound in plasma (95.3%) with some binding to albumin (4.2%) and at a lower extent to HDL (0.5%), indicating that PAs bind to plasma components through their lipid modifications.

To further study the plasma components that PAs bind, mass spectrometry (MS) was used. For MS experiments, we only used SA‐E due to its effective assembly with plasma components. A biotinylated version of ICG labeled SA‐E was prepared (SA‐Eb; Figure S11, Supporting Information) and incubated in human plasma. SA‐Eb and the plasma components bound to it were pulled down using streptavidin‐coated magnetic beads, and the eluted proteins were analyzed using MS (Figure 1f). There were ≈2.4‐fold more MS counts for the SA‐Eb sample compared to proteins eluted from beads in the absence of SA‐Eb (only plasma), suggesting that most proteins were specifically pulled down with SA‐Eb (Table S1, Supporting Information). For the SA‐Eb sample, 28.5% of all detected proteins were apolipoproteins (protein components of LPs), whereas the apolipoprotein content was 11.8% in the plasma only sample (Figure 1f; Table S1, Supporting Information). Apolipoprotein B, the main protein component of LDL and VLDL,^[^ ^46^ ^]^ was non‐detectable in the plasma only sample, but it was abundant (5.4%) in the presence of SA‐Eb. In addition, the concentration of Apolipoprotein A‐I, the main protein component of HDL,^[^ ^46^ ^]^ increased ≈2.5‐fold (to 18.4% from 7.3%) in the SA‐Eb sample. The increase in the albumin concentration (to 33.3% from 31.8%) was lower than the increase in LPs, which is in accordance with FPLC results. Overall, these results further suggested that SA‐E preferentially assembles with LPs in the blood.

### 2.2 Assembly of PAs With Endogenous Blood Biomolecules Prolongs Circulation

We next explored the interactions of PAs with blood components in vivo to confirm that they interact with LPs in situ upon systemic delivery. For these experiments, we used SA‐E due to its better binding ability to plasma components. ICG conjugated SA‐E was intravenously injected into wild type mice, and blood samples were collected 1 h after SA‐E injection. FPLC of plasma (Figure 1g) showed that most of SA‐E was most preferentially bound to HDL, again with similar percentages observed in human plasma (LPs: 65.8% total, 55.2%, 4.6%, and 5.9% for HDL, LDL, and VLDL, respectively, and albumin: 34.2%). We also measured the ICG fluorescence in fractions of collected blood samples: plasma, white blood cells (WBC), and red blood cells (RBCs), to understand if SA‐E is also assembled or taken up by blood cells (Figure S12, Supporting Information). On average, 98.9% of SA‐E fluorescence was detected in the plasma, with only 1.1% fluorescence in the RBC component, suggesting that SA‐E almost completely assembles with plasma components in the blood, and its uptake by blood cells is negligible.

We then utilized two genetically engineered mouse models with aberrant lipid metabolism, ApoA1 and LDL‐R knockout mice (ApoA1‐/‐ and LDL‐R‐/‐), to further investigate LP specific binding of SA‐E in blood and understand how the altered lipid metabolism affects interactions of SA‐E with LPs. As SA‐E mainly binds to HDL in the blood, we first used ApoA1‐/‐ mice, which have significantly reduced levels of HDL due to the lack of the main apolipoprotein component of HDL in these mice.^[^ ^47^ ^]^ FPLC showed a dramatically reduced HDL binding (16.7%) in ApoA1‐/‐ mice compared to wild type mice with significantly increased albumin binding (75.4%), confirming that SA‐E mainly assembles with HDL in circulation (Figure 1h). We then utilized LDL‐R‐/‐ mice, which have elevated blood cholesterol and LPs, especially LDL,^[^ ^48^ ^]^ to explore how increased cholesterol levels affect the interactions of SA‐E with plasma components. In general, a similar binding profile with wild type mice was observed for LDL‐R‐/‐ mice (Figure 1h) with slightly increased overall LP binding from 65.8% to 73%. A more significant difference was the increase in SA‐E assembly with LDL and VLDL. While the LDL and VLDL were not separated as observed for wild type mice, total binding to these LPs increased to 28.6% from 10.6%. Nevertheless, SA‐E was found to be mostly assembled with HDL (44.4%), showing that even a dramatic change in cholesterol levels does not significantly affect SA‐E assembly with blood components.

After confirming the preferential assembly of PAs with LPs, especially HDL, we investigated how LP binding affects the blood circulation of PAs in wild‐type mice. SA‐E showed prolonged blood circulation compared to both SA‐K and free ICG (Figure 1i). 21% of the injected SA‐E was still in circulation 6 hours post injection compared to 5% of SA‐K. In addition, the AUC of the blood circulation plot was 2.1‐fold higher for SA‐E than AUC of SA‐K. Blood concentration of free ICG quickly decreased below 1% of the injected dose in 30 min, and it was not detectable 6 h after injection, which is in accordance with the previous literature.^[^ ^49^ ^]^ We also studied the blood circulation of ICG labeled No‐SA to further explore the effect of LP binding on blood circulation (Figure S13, Supporting Information). While No‐SA showed improved blood circulation compared to free ICG, it cleared more rapidly compared to SA‐E, with only ≈2.5% of the injected dose remaining in circulation 6 h after injection. Altogether, these in vitro and in vivo experiments showed that SA‐E nanostructures can effectively disassemble and reassemble with endogenous blood biomolecules in circulation, which prolongs blood circulation compared to structurally more stable assemblies of SA‐K or non‐LP binding No‐SA.

### 2.3 Cellular Entry of PAs

Next, we investigated interactions of PAs with cell membranes and the impact of LPs on these interactions using Cy5 labeled PAs (Figures S14 and S15, Supporting Information). First, to understand if replacing the ICG with Cy5 affected the morphology and structural stability of PA micelles, we performed TEM and fluorescence measurements. TEM imaging showed spherical and rod‐like micelle formation for SA‐E and SA‐K, respectively, with similar morphologies observed for ICG labeled PAs (Figure S16, Supporting Information). Fluorescence measurements showed that while there was a weak fluorescence in the SA‐E sample, Cy5 fluorescence was mostly quenched for both PAs (Figure S17, Supporting Information). When incubated in 10% human plasma for 1 h, the increase in Cy5 fluorescence was ≈10‐fold higher in the SA‐E sample compared to the SA‐K, which suggested a higher stability for the SA‐K micelles. Overall, Cy5 conjugation did not significantly affect the assembly and structural stability of SA‐E and SA‐K micelles compared to their ICG conjugated counterparts.

**Figure 2:**
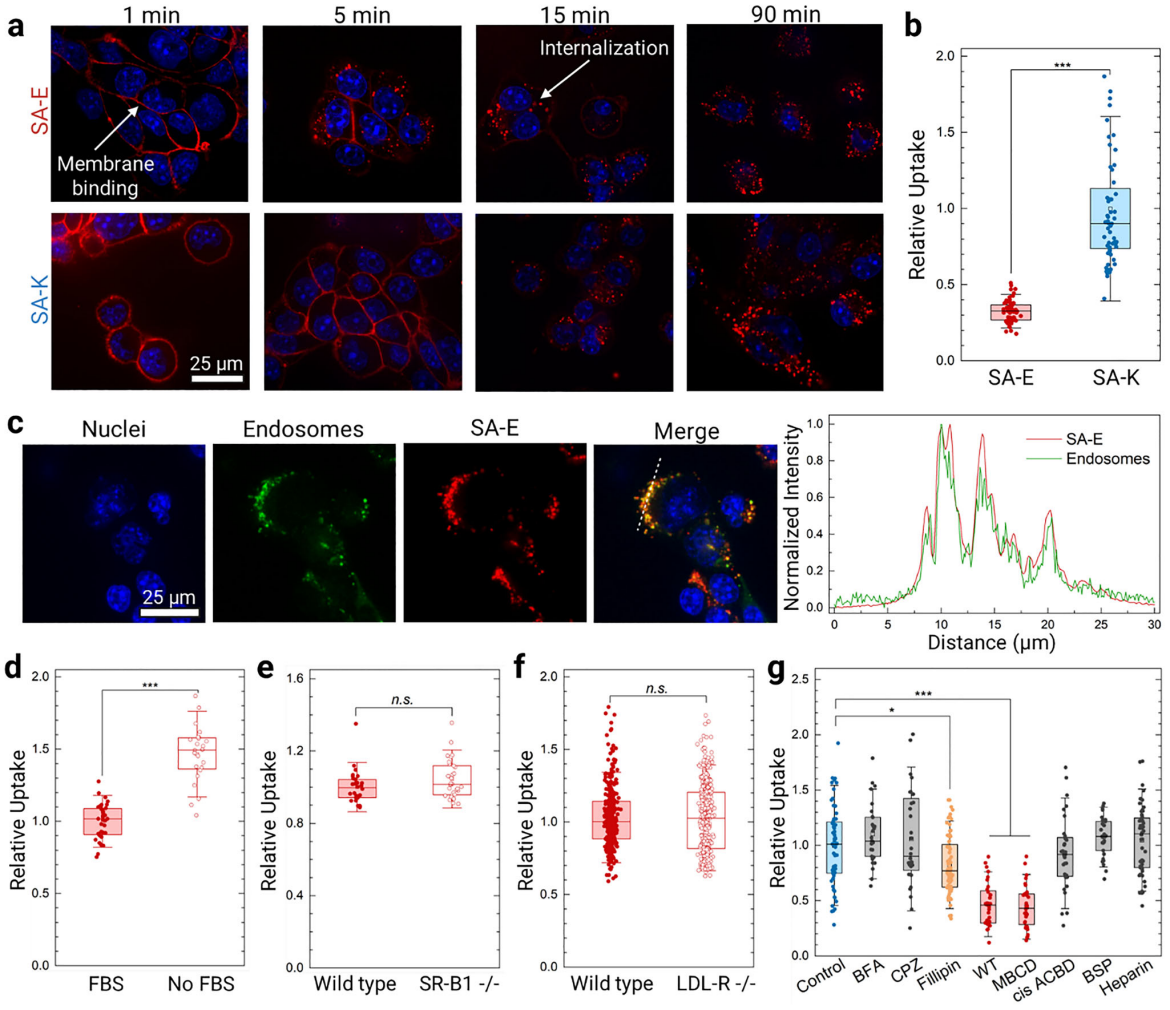
PAs internalize into cells through binding to lipid raft domains of cell membranes. a) Confocal microscope images of 4T1 cells incubated with SA‐E or SA‐K at different time points. b) Uptake of PAs by 4T1 cells. c) Confocal images of 4T1 cells incubated with endosomal stain (FITC‐dextran) and Cy5 labeled SA‐E. The panel on the right shows the intensity profile of FITC and Cy5 through the dashed line in the merged image. d) Uptake of SA‐E by 4T1 cells in the presence or absence of FBS. e) Uptake of SA‐E by wild type or SR‐B1 knock out TRAMP‐C2 mouse prostate cells. f) Uptake of SA‐E by wild type or LDL‐R knock out HeLa human cervical cancer cells. g) Uptake of SA‐E by 4T1 cells in the presence of different inhibitors. BFA is Brefeldin A, CPZ is Chlorpromazine, WT is Wortmannin, MBCD is Methyl‐β‐cyclodextrin, and BSP is Bromosulfalein. The whisker box plots in (b,d‐g) show the 25th and 75th percentiles with the median represented by the center line. Whiskers extend to 1.5x the inter‐quartile ranges (IQR). Statistical analysis was performed using one‐way analysis of variance (ANOVA) in (g) and Student's *t*‐test in (b,d‐f). *n.s*. is non‐significant, ^*^ *p* < 0.05 and ^***^ *p* < 0.001.

We then incubated PAs briefly (≈1 min) with 4T1 mouse breast cancer cells and imaged the cells at different time points after removing the PAs. For both SA‐E and SA‐K, a strong Cy5 fluorescence was observed at the cell membrane shortly after adding the PAs (**Figure** **2**a). To confirm membrane binding of PAs, we used a membrane stain (CellBrite 488), which showed a good overlap between the Cy5 signal of PAs and the membrane stain at 5 min (Figure S18, Supporting Information). Further incubation resulted in the internalization of PAs into cells (Figure 2a; Figure S18, Supporting Information). While both SA‐E and SA‐K were effectively taken up by cells, SA‐K showed approximately three‐fold higher uptake than SA‐E (Figure 2b), which might be due to its positive charge.^[^ ^50^ ^]^ As fluorescence of Cy5 was mostly quenched in the intact PA micelles, the signal on the cell membrane at initial time points suggests fluorescence recovery through disassembly and binding to cell membranes, which is also in accordance with previous reports.^[^ ^40^, ^51^, ^52^ ^]^ In addition, no significant membrane binding or internalization was observed for Cy5‐conjugated No‐SA (Figures S19 and S20, Supporting Information), showing that lipid modification of PAs is required for their cellular internalization. Nevertheless, as the fluorescence of micelles prepared using 100% of Cy5‐conjugated PAs is mostly quenched, the experiments above only show the uptake of disassembled PA monomers and may not detect the potential uptake of intact micelles. Therefore, we also performed uptake experiments using fluorescent micelles, which were prepared by reducing the Cy5 amount in the micelles to 10%, as described above. For these experiments, we blocked cysteine residues of unconjugated PAs using maleimide to prevent them from interacting with free thiols (Figures S21 and S22, Supporting Information). 10% Cy5 micelles showed similar uptake behavior to 100% micelles (Figure S23, Supporting Information) with initial membrane binding followed by internalization. We also incubated the cells with 10% and 100% Cy5 PAs for 2 h, and quantified the total uptake (Figure S24, Supporting Information). While no significant difference in the uptake of between 10% and 100% Cy5 micelles was observed for SA‐E, uptake of 10% micelles was ≈1.5‐fold higher than 100% micelles for SA‐K. All together, these results suggested that weakly assembled SA‐E micelles disassemble in cell culture conditions and internalize into cells by binding cell membranes through their lipid tails. For more stable SA‐K micelles, while uptake through membrane binding of their monomers is also a major internalization mechanism, some uptake as intact micelles also occurs. To understand if disassembled PA micelles enter cells through passive diffusion or endosomal pathways, we incubated cells with SA‐E and an endosome stain (fluorescein isothiocyanate conjugated dextran, FITC‐dextran) for 4 h. Fluorescence of SA‐E and endosomal fluorescein showed a good correlation with a Pearson coefficient of 0.605 ± 0.02 (Figure 2c; see also Video S1, Supporting Information), suggesting that SA‐E was internalized in cells mainly via endosomal uptake.

Next, we explored the effect of LPs and albumin on the cellular uptake of PAs using SA‐E. First, we studied the uptake of SA‐E by 4T1 cells in the presence or absence of blood biomolecules by using cell media with or without fetal bovine serum (FBS). The absence of FBS in cell media did not decrease SA‐E uptake. On the contrary, SA‐E uptake was ≈1.6‐fold higher in FBS‐free media (Figure 2d), which suggests a competitive binding of SA‐E with FBS biomolecules and cell membranes that can decrease the uptake. In addition, we studied the impact of two well‐known LP receptors for LDL and HDL, LDL‐R and Scavenger receptor B type 1 (SR‐B1), respectively,^[^ ^33^, ^53^ ^]^ on SA‐E uptake using cells knockout for these receptors. We did not observe any significant change in the cellular uptake of SA‐E in LDL‐R knockout (LDL‐R‐/‐) HeLa cervical or SR‐B1 knockout (SR‐B1‐/‐) TRAMP‐C2 mouse prostate cancer cells compared to wild type controls (Figure 2e,f).^[^ ^54^ ^]^

To further evaluate the uptake mechanism of SA‐E, we used a series of inhibitors blocking different endocytosis pathways or cell surface receptors (Figure 2g).^[^ ^55^, ^56^, ^57^, ^58^ ^]^ Significant decreases in the cellular uptake were observed for Wortmannin (WT, a PI3K inhibitor and general inhibitor of endocytosis) and Methyl‐β‐cyclodextrin (MBCD, extracts cholesterol from cell membranes). In addition, Filipin, a caveolae‐dependent endocytosis inhibitor and a binder of membrane cholesterol,^[^ ^59^ ^]^ decreased SA‐E uptake to a lesser extent. As cholesterol is enriched in lipid rafts, which are solid domains of cell membranes,^[^ ^60^, ^61^, ^62^ ^]^ these results indicate a higher affinity of SA‐E toward cholesterol‐rich lipid‐raft domains of cell membranes. Since SA‐E contains several anionic residues, we also used inhibitors of glutamate uptake (cis‐ACBD) or organic anionic uptake (bromosulfalein, BSP)^[^ ^63^, ^64^ ^]^ and found no change in SA‐E uptake. Altogether, these results suggest that PAs are internalized in cancer cells via membrane binding, especially to cholesterol‐rich domains, independent of LP or other cell surface receptors.

### 2.4 Dynamic Interactions of PAs With Endogenous Biomolecules Improve Tumor Accumulation

Next, we studied the biodistribution of PAs in tumor‐bearing mice to understand how their interactions with plasma components and cell membranes affect their accumulation in solid tumors and healthy tissues. As discussed above, intact micelles of PAs are not fluorescent. Thus, fluorescence imaging can only detect disassembled PAs. Therefore, we first investigated the disassembly of PAs in vivo by incubating them in mouse liver homogenates. SA‐E and SA‐K showed almost identical fluorescence intensity in liver homogenates (Figure S25, Supporting Information), indicating a similar disassembly degree for SA‐E and SA‐K in the tissues and confirming that fluorescence imaging using an in vivo imaging system (IVIS) could be used to compare biodistribution of these two PAs with different plasma stability.

**Figure 3:**
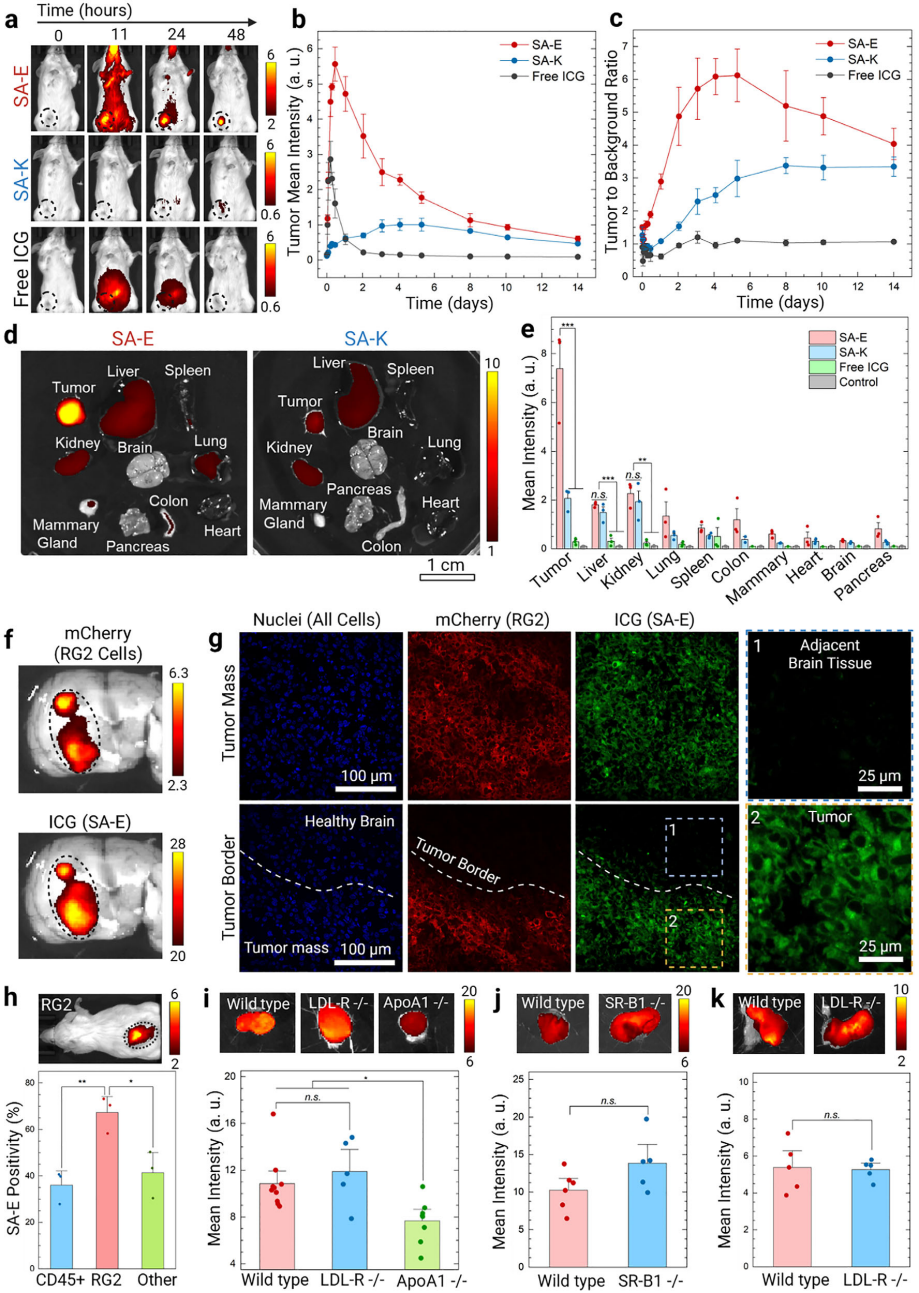
SA‐E shows strong tumor accumulation and retention. a) Representative IVIS images of intravenously injected ICG labeled SA‐E or SA‐K (50 nmol) or free ICG in 4T1 tumor‐bearing mice at different time points. Black circles highlight the tumor location. b) Calculated mean intensities of ICG signal and c) tumor to background signal ratio of SA‐E, SA‐K, and free ICG at different time points after injection. d,e) Biodistribution of SA‐E, SA‐K, or free ICG in 4T1 tumor‐bearing mice. d) Representative IVIS images showing accumulation of SA‐E and SA‐K in tumors and major organs. Tissues were excised 2 days after injection. e) Mean ICG fluorescence intensity in the tumor and major organs of mice received SA‐E, SA‐K, or free ICG injection compared to control mice. f) IVIS imaging of mCherry expressing RG2 tumor sections showing a good overlap between the mCherry signal of RG2 cells and the ICG signal of SA‐E. g) Confocal microscope images of a brain tissue section with mCherry expressing RG2 tumors showing specific accumulation of SA‐E in RG2 tumors and internalization in cells in the tumor microenvironment. h) Flow cytometry analysis of mCherry expressing RG2 xenografts in mice that received SA‐E injection (50 nmol) 2 days before harvesting tumors. The top panel is an IVIS image of RG2 tumor‐bearing mice showing accumulation of SA‐E in the tumor. i) Mean tumor intensity of SA‐E 2 days after injection (50 nmol) into MC‐38 tumor‐bearing wild type, LDL‐R‐/‐, or ApoA1‐/‐ mice. Images on the top are representative IVIS images of the tumors. j) Mean tumor intensity of SA‐E 2 days after injection (50 nmol) into wild type or SR‐B1‐/‐ TRAMP‐C2 tumor‐bearing mice. Images on the top are representative IVIS images of the tumors. k) Mean tumor intensity of SA‐E 2 days after injection (50 nmol) into wild type or LDL‐R‐/‐ HeLa tumor‐bearing mice. Images on the top are representative IVIS images of the tumors. Data are presented as mean ± SEM. Studies were run in at least triplicates, except for ICG accumulation in the colon, mammary gland, heart, brain, and pancreas in (e) was obtained using a single mouse, and SA‐K accumulation in the colon in (e) was obtained using two mice. Statistical analysis was performed using one‐way analysis of variance (ANOVA) in (e, h, and i) and Student's t test in (j and k). *n.s*. is non‐significant, ^*^ *p* < 0.05, ^**^ *p* < 0.01, and ^***^ *p* < 0.001.

We initially evaluated the tumor accumulation of ICG conjugated SA‐E and SA‐K (50 nmol, intravenously injected) within a syngeneic mouse 4T1 breast cancer model. We also used a free ICG injection as a control. IVIS imaging showed a significantly higher tumor accumulation for SA‐E compared to both SA‐K and free ICG (**Figure** **3**a). For SA‐E, maximum fluorescence levels were detected in the tumor at 11 h post‐injection (Figure 3b). SA‐E signal slowly decreased over 2 weeks with a 50% decrease in the signal on day 3, and still detectable levels even 2 weeks after injection, demonstrating its prolonged retention. Background signal was mostly cleared 2 days after injection (Figure 3a), and tumor to background signal ratio (TBR) remained above 2.5 from 11 h to 2 weeks post‐injection with a maximum of around 5–6 from days 2 to 8 (Figure 3c). SA‐K, with better stability in circulation, reached a maximum at 4 days post‐injection, and it was ≈6‐fold lower than the maximum signal of SA‐E. Also, the total tumor accumulation of SA‐K was ≈2.7‐fold lower than SA‐E (Figure S26, Supporting Information). In addition, the maximum TBR was approximately two‐fold lower than that of SA‐E. Free ICG did not identify the tumor at any time point, and its TBR ratio always remained around 1. We also evaluated the tumor accumulation of ICG conjugated No‐SA to understand the effect of LP binding on cancer targeting (Figure S27a,b, Supporting Information). ICG conjugated to No‐SA showed significantly improved tumor accumulation than free ICG, which can be explained by the improved blood circulation of No‐SA. Compared to SA‐E, No‐SA accumulated more quickly in the tumor and mostly cleared in 2 days. Total tumor accumulation of SA‐E was ≈1.6‐fold higher than No‐SA (Figure S26, Supporting Information). In addition, No‐SA showed significantly lower TBR than SA‐E (Figure S27c, Supporting Information).

Next, we investigated the biodistribution of PAs and free ICG in Balb/c mice bearing 4T1 tumors by imaging the organs 2 days post‐injection (50 nmol). In line with live animal imaging, a significantly stronger fluorescence in the tumor was observed for SA‐E compared to SA‐K and free ICG (Figure 3d,e; Figure S28, Supporting Information). Notably, SA‐E demonstrated a high tumor‐to‐liver signal ratio of 4.1, whereas it was 1.4 for SA‐K. To estimate the percent injected dose (%ID) in each organ, tumors and major organs were homogenized, and ICG fluorescence in each organ was measured (Figure S29, Supporting Information). In accordance with IVIS imaging, SA‐E concentration in the tumor (1.9% ± 0.4%ID / g tissue) was significantly higher than liver (0.8% ± 0.1%ID / g tissue) and other major organs. Finally, to compare the biodistribution of SA‐E and SA‐K micelles that are fluorescent before disassembling in vivo, we reduced the ICG concentration to 10% using maleimide blocked PAs, as described above. Similar tumor accumulation and biodistribution in 4T1 tumor‐bearing mice were observed for the 10% ICG micelles (Figure S30, Supporting Information) compared to their 100% ICG counterparts described above. While it is known that physicochemical properties (e.g., surface charge, shape, and stiffness) of nanoparticles have a significant impact on their biodistribution,^[^ ^65^, ^66^, ^67^ ^]^ our findings suggested that for the PAs used in this study, an important factor determining their biodistribution is their ability to disassemble in circulation and reassemble with plasma components. Due to its superior tumor accumulation and biodistribution, we used SA‐E in the rest of the study.

We studied the clearance pathway and toxicity of SA‐E in wild type mice. For clearance studies, SA‐E (50 nmol) was injected into wild type mice, and urine and stool samples were collected at different time points (Figure S31, Supporting Information). A strong ICG signal was detected in the stool samples, which peaked at 6 h after injection and mostly diminished at 2 days. No fluorescence signal was detected in urine samples at any time point. These results indicated that SA‐E was cleared through liver and intestines as expected for LPs.^[^ ^68^ ^]^ The toxicity of SA‐E was studied in wild type mice using a four‐fold higher dose than the optimal imaging dose (200 nmol). We measured complete blood counts (CBC) for blood toxicity, Aspartate transferase (AST) for liver toxicity, and Creatinine for kidney toxicity from blood samples collected 1 day after SA‐E injection and found no difference between SA‐E or control, suggesting SA‐E is highly biocompatible (Figure S32, Supporting Information).

To further evaluate tumor‐specific accumulation and in vivo cancer cell internalization of SA‐E, we next utilized an orthotopic rat RG2 glioblastoma model. For these experiments, RG2 cells were injected into the rat cerebrum, and their growth was followed using gadolinium contrast‐enhanced magnetic resonance imaging (MRI). ICG labeled SA‐E (500 nmol) was injected when the tumors were easily visualized by MRI. Figure S33a (Supporting Information) shows the distribution of SA‐E in the organs of a RG2 tumor‐bearing rat, where significant ICG fluorescence was observed in the RG2 cell injection site with significantly lower accumulation in major organs. To better image the tumors, brains were wholemount bisected through the approximate middle of the tumor in a similar orientation as the MRI. There was near complete overlap between the T1 MRI signal and SA‐E signal (Figure S33b, Supporting Information). Overall, the SA‐E signal in the tumor was ∼16‐fold higher than the adjacent brain tissue (Figure S33c, Supporting Information). To confirm the specific accumulation of SA‐E in cancer cells, we used mCherry expressing RG2 cells. IVIS imaging of a wholemount bisected brain sample showed an almost complete overlap between the mCherry fluorescence of RG2 cells and ICG fluorescence of the SA‐E (Figure 3f). Confocal microscopy showed cellular internalization of SA‐E in RG2 cells and uniform accumulation in the tumor tissue with clear tumor margins and no detectable fluorescence in the healthy brain tissue (Figure 3g).

We used flow cytometry to explore if SA‐E is taken up more by cancer cells compared to other cell types in the tumor microenvironment due to increased lipid uptake of cancer cells.^[^ ^33^ ^]^ For these studies, we used mCherry expressing RG2 xenografts in mice, and Cy5 conjugated SA‐E. We first confirmed the accumulation of SA‐E in RG2 tumors using ICG labeled SA‐E (Figure 3h). Then, Cy5 labeled SA‐E was injected into tumor‐bearing mice, tumors were harvested 2 days after SA‐E (50 nmol) injection, and flow cytometry was performed for RG2 (mCherry), white blood, and other cells in the tumor stroma. mCherry expressing cancer cells also containing Cy5 were found ≈70% of the time, whereas CD45 positive only (white blood cells) or CD45 and mCherry negative cells (tumor stroma) contained Cy5 ≈35%–40% of the time (Figure 3h; Figure S34, Supporting Information). These results demonstrated that SA‐E can effectively internalize in cancer cells and is enriched (approximately two‐fold) in cancer cells compared to other cells in the tumor environment.

**Figure 4:**
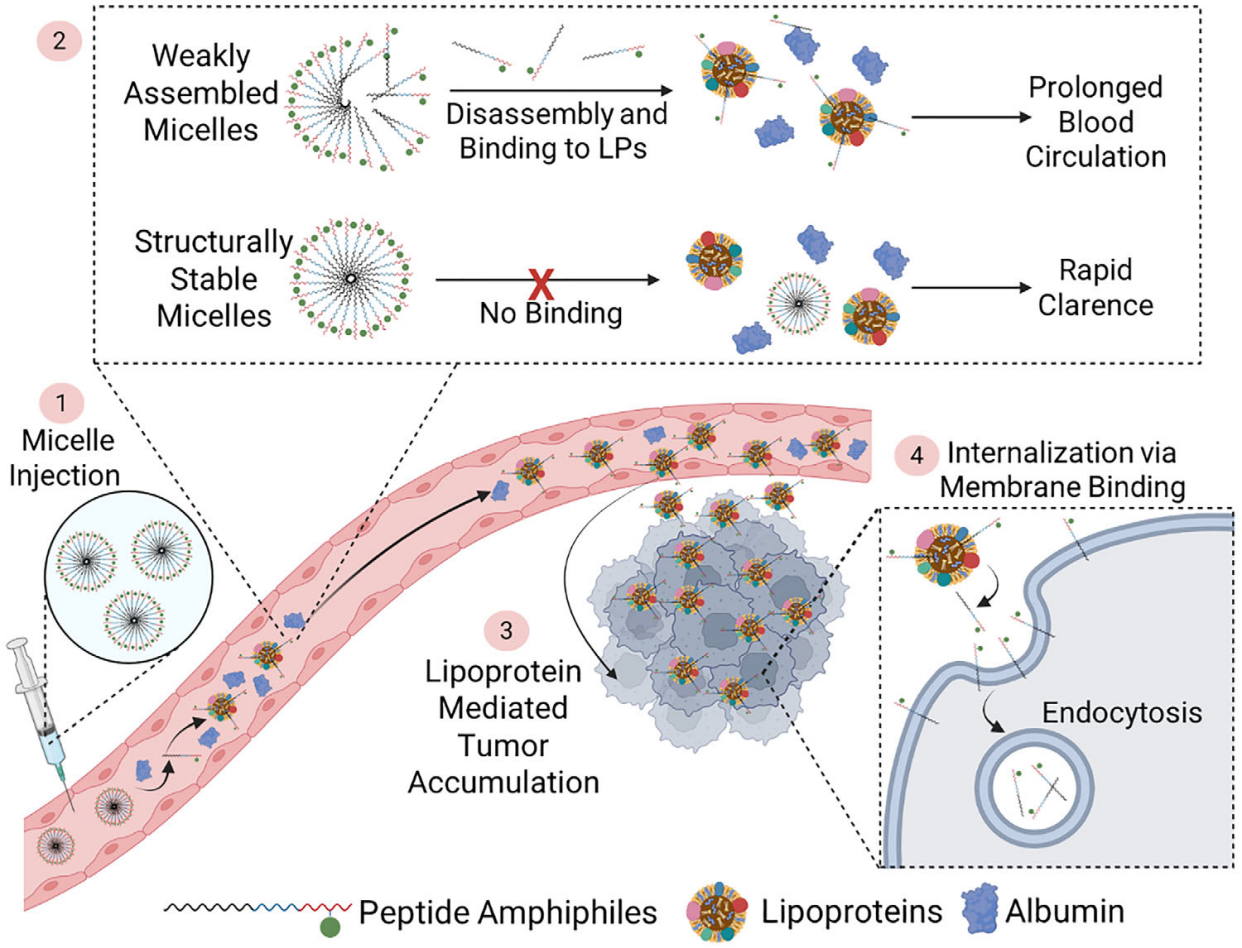
Schematic showing the proposed tumor accumulation and cancer cell internalization mechanisms of SA‐E. Created with Biorender.com.

Next, we performed in vivo experiments using different knockout strategies to study the proposed LP hitchhiking based tumor accumulation mechanism of PAs. First, we studied the tumor accumulation of SA‐E in syngeneic MC38 colon cancer tumors in wild type, ApoA1‐/‐, or LDL‐R‐/‐ mice (Figure 3i). Compared to the tumors formed in wild type mice, SA‐E uptake was significantly lower in ApoAI ‐/‐ mice, suggesting that assembly with HDL is critical for SA‐E to accumulate in solid tumors. On the other hand, there was no significant change in LDL‐R‐/‐ mice, indicating that alterations in the cholesterol levels do not significantly affect the tumor targeting of SA‐E. In addition, SA‐E accumulation in other organs was not significantly different from wild type control for both models (Figure S35, Supporting Information). Next, we wanted to understand the role of receptor‐mediated uptake in the cancer cells in vivo using SR‐B1 knockout TRAMP‐C2 mouse prostate cancer and LDL‐R knockout human cervical cancer HeLa cells with their wild type controls (Figure 3j,k). In accordance with cell culture results, SR‐B1 or LDL‐R was not needed for tumor accumulation. Overall, these results suggest a mechanism of dynamic assembly to LPs in circulation followed by another dynamic assembly for a non‐receptor mediated cancer cell uptake that occurs in vivo and is supported by our initial in vitro experiments. The proposed mechanism for cancer‐specific accumulation of PA nanostructures with low structural stability is summarized in **Figure** **4**. In summary, weakly assembled PAs quickly disassemble and bind to plasma biomolecules in circulation, primarily HDL, but also to other LPs and albumin to a lower extent. Assembly with these biomolecules improves the blood circulation of PAs and allows their effective accumulation in solid tumors.^[^ ^32^, ^33^, ^34^, ^35^, ^42^, ^43^, ^44^, ^45^, ^46^ ^]^ In the tumor microenvironment, they assemble with cell membranes, mainly lipid‐raft domains, for cellular internalization through endosomal uptake.

### 2.5 SA‐E Accumulated in a Broad Range of Solid Tumors

**Figure 5:**
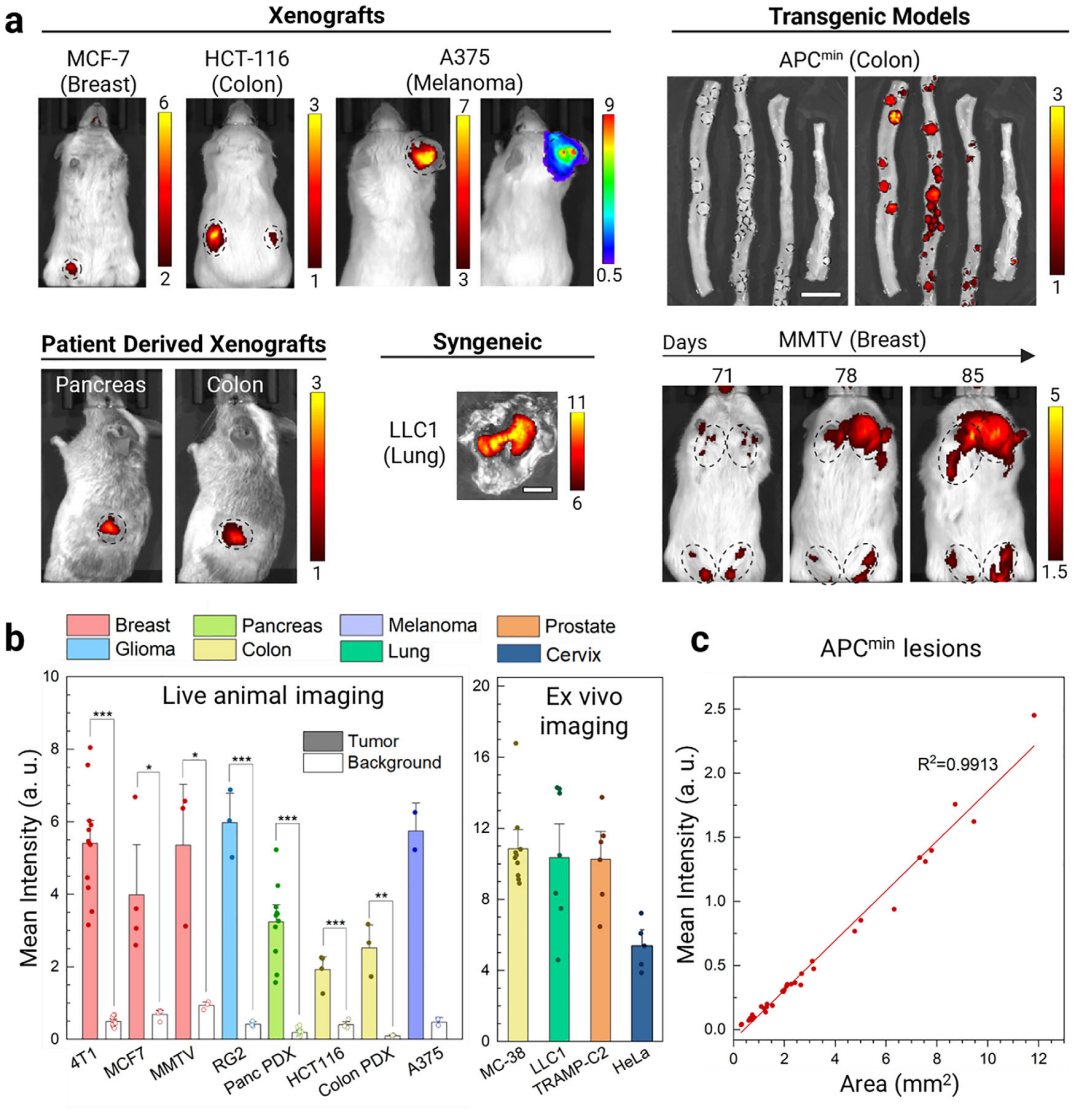
SA‐E accumulates in a broad range of solid tumors. a) Representative IVIS images showing the SA‐E accumulation in various tumor models. MCF7, A375, and HCT‐116 are human cell lines. LLC1 is a mouse cell line. In addition, two patient‐derived xenografts (PDXs) of human colon and pancreatic cancer were used. MMTV and APC^min^ are transgenic mouse models of breast and colon cancer, respectively. For the MMTV model, mice were imaged weekly. For the APC^min^ model, bright field (left) and fluorescence (right) IVIS images show small intestine and colon samples of an APC^min^ mouse injected with SA‐E at 4 months of age. IVIS imaging was performed 2 days after ICG labeled SA‐E injection (50 nmol), either using live animals or excised tumors. For the luciferase‐expressing A375 model, luciferase imaging was also performed (right panel), showing a good overlap between ICG and luciferase signals. Black circles highlight the areas with tumors, adenomas or polyps. Scale bars are 1 cm. b) Tumor and background intensity of SA‐E in different models: live animal imaging (left) and *ex vivo* imaging (right). c) Correlation between lesion size and SA‐E signal in the lesions detected in the small intestines and colons of three Apc^min^ mice. Data are presented as mean ± SEM. Studies were run in at least triplicates except the A375 model, which was performed in duplicate. Statistical analysis was performed using Student's *t* test. ^*^ *p *< 0.05, ^**^ *p *< 0.01 and ^***^ *p *< 0.001. No statistical analysis was performed for the A375 model.

Since the proposed tumor‐targeting mechanism of SA‐E is universal, we tested tumor accumulation in a broad range of cell types and tissue locations. In addition to the five tumor models discussed above, we utilized 8 additional xenograft, syngeneic, transgenic, and PDX tumor models in mice. SA‐E demonstrated highly specific tumor accumulation in all 13 tumor models of 8 different tissue types (**Figure** **5**a). The tumors used in these studies ranged in size from a few millimeters to a centimeter in syngeneic models in immune competent mice, as well as human or rat cells in immunocompromised mice. Strong tumor signal and high tumor‐to‐background ratios ranging between ≈3.5 and 50 were observed for the tumors imaged in live animals (Figure 5b). Also, by utilizing the A375 human melanoma cell line expressing luciferase, we showed that the SA‐E signal overlapped with where the tumor was located. In addition, SA‐E demonstrated strong accumulation in 2 transgenic models: MMTV‐PyMT and APC^min^. MMTV‐PyMT mice develop early to late cancer lesions in all mammary glands with time.^[^ ^69^ ^]^ Figure 5a shows representative images from an MMTV‐PyMT mouse injected with SA‐E weekly up to 85 days of age when all mammary glands developed tumors with different sizes. Notably, SA‐E accumulated in early small lesions at 71 days of age, which developed large tumors 1–2 weeks later. In addition, organs harvested at the end of the experiment showed strong accumulation in mammary tumors but not in other organs (Figure S36, Supporting Information). APC^min^ mice are a transgenic colon cancer model suitable for studying early adenoma lesions.^[^ ^70^ ^]^ Small intestinal adenomas and colon polyps of APC^min^ mice injected with SA‐E at 4 months of age had higher fluorescent signals than the surrounding normal intestine tissue (Figure 5a). Overlaying the identified tumors with SA‐E fluorescence signal revealed that 100% of small intestinal adenomas and colon polyps were positive. In total, 71/71 small intestinal adenomas and 4/4 colon polyps were detected in 3 mice (Table S2, Supporting Information) by marking all adenomas and polyps using the photograph images. In addition, a good linear correlation between lesion size and SA‐E signal was observed with tumors even smaller than 1 mm^2^ being identified (Figure 5c). These results strongly suggest that SA‐E uses a universal mechanism to target a broad range of tumors, including millimeter‐sized early tumor lesions.

### 2.6 SA‐E Enables More Efficient and Safer Chemotherapy

Based on strong tumor accumulation and retention of SA‐E, we hypothesized that SA‐E can improve the efficacy of chemotherapies and decrease their side effects. While in previous studies, PAs have been applied for drug delivery to cancer and other diseases, in these studies, drugs were typically loaded into the hydrophobic cores of the PA assemblies.^[^ ^71^, ^72^, ^73^, ^74^, ^75^ ^]^ As SA‐E quickly disassembles in circulation and is transported to solid tumors as monomers, instead of physical loading, we chemically conjugated a chemotherapy agent to SA‐E using a cleavable linker. It should also be noted that the chemical conjugation strategy brings several advantages over physical loading, such as minimal drug leakage in circulation and suitability for reproducible and large‐scale production. Specifically, we conjugated a highly potent tubulin inhibitor, Monomethyl auristatin E (MMAE), using an MMAE prodrug (vcMMAE) with a Cathepsin B cleavable linker (Figure S37, Supporting Information).^[^ ^76^ ^]^ TEM imaging of SA‐E‐MMAE micelles showed the formation of spherical micelles (Figure S38, Supporting Information). To evaluate if conjugation of hydrophobic MMAE to SA‐E affects the intramolecular interactions in SA‐E micelles, we performed fluorescence polarization measurements using SA‐E micelles prepared by mixing ICG‐conjugated SA‐E (10 mol%) with MMAE‐conjugated SA‐E (90 mol%). While the anisotropy of ICG in SA‐E micelles increased slightly from 0.123 ± 0.006 to 0.188 ± 0.002 in the presence of 90% MMAE, suggesting an increase in the rigidity of SA‐E micelles,^[^ ^18^ ^]^ fluorescence anisotropy was significantly lower than that of stable SA‐K micelles, 0.299 ± 0.008. FPLC was utilized to evaluate the stability of SA‐E‐MMAE micelles in human plasma, as described above. For FPLC measurements, the strong absorbance of MMAE at ≈250 nm,^[^ ^77^ ^]^ where plasma has a weak absorbance (Figure S39a, Supporting Information), was used to detect SA‐E‐MMAE. In PBS, SA‐E‐MMAE micelles were eluted as a single peak (Figure S39b, Supporting Information) similar to the results obtained for ICG conjugated SA‐E micelles (Figure 1d). In human plasma, FPLC showed that SA‐E‐MMAE assembled with HDL as observed for ICG conjugated SA‐E (Figure S39c, Supporting Information). These results suggested that conjugating MMAE to SA‐E did not significantly affect its assembly and disassembly in circulation.

**Figure 6:**
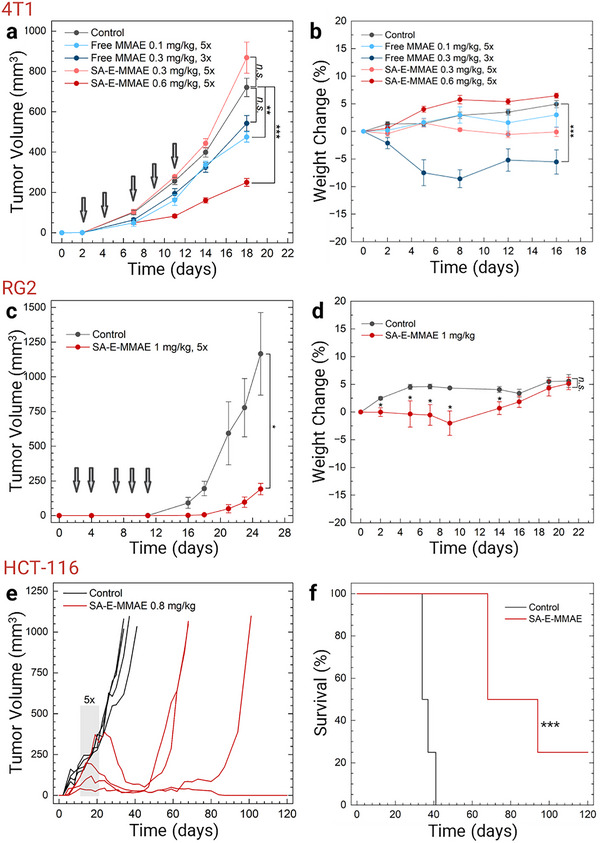
SA‐E improves the antitumor efficacy and safety of MMAE. a) Tumor volume of 4T1 tumor bearing mice received PBS (*n* = 18), 0.1 mg kg^−1^ of free MMAE (*n* = 3), 0.3 mg kg^−1^ of free MMAE (*n* = 5), 0.3 mg kg^−1^ of SA‐E‐MMAE (*n* = 5), or 0.6 mg kg^−1^ of SA‐E‐MMAE (*n* = 5). All mice received 5 doses of PBS, free MMAE, or SA‐E‐MMAE except 0.3 mg kg^−1^ free MMAE group, which received first 3 doses due to significant weight loss. b) Average body weight change of the mice that received the treatments in (a). c) Tumor volume of RG2 tumor bearing mice received 5 doses of intravenous injection of PBS (*n* = 4) or 1 mg kg^−1^ of SA‐E‐MMAE (*n* = 4). d) Average body weight change of the mice that received the treatments in (d). e) Tumor volume of HCT‐116 tumor bearing mice received 5 doses of intravenous injection of PBS (*n* = 4) or 0.8 mg kg^−1^ of SA‐E‐MMAE (*n* = 4) on days 11, 14, 17, 19, and 21. f) Kaplan‐Meier survival plot of the mice in (e). Grey arrows in (a) and (c) and grey box in (e) show the days that the mice received PBS or drug injections. Data are presented as mean ± SEM. Statistical analysis was performed using two‐way analysis of variance (ANOVA) in (a) and (b), Student's *t*‐test in (c) and (d), and log‐rank test in (f). *n.s*. is non‐significant, ^*^ *p* < 0.05, ^**^ *p* < 0.01, and ^***^ *p* < 0.001.

SA‐E‐MMAE inhibited the growth of 4T1 and RG2 cells in vitro with IC50 values of 1.18 and 0.21 µM, respectively (Figure S40, Supporting Information). We then tested SA‐E‐MMAE in the 4T1 orthotopic mouse model. 4T1 tumor bearing mice were intravenously injected with 5 doses of free MMAE (0.1 or 0.3 mg kg^−1^) or SA‐E‐MMAE (0.3 or 0.6 mg kg^−1^, based on MMAE) over 11 days. Free MMAE injections resulted in a slight reduction in tumor growth (**Figure** **6**a). While free MMAE was tolerated at 0.1 mg kg^−1^, increasing the dose to 0.3 mg kg^−1^ resulted in high toxicity with rapid weight loss, and treatment needed to be stopped after the third injection (Figure 6b). SA‐E‐MMAE was tolerated much better at higher doses. There was no significant weight loss at a two‐fold higher dose of 0.6 mg kg^−1^ with SA‐E‐MMAE. Importantly, SA‐E‐MMAE showed significant tumor growth reduction (≈75%) compared to both free MMAE and PBS control tumors at the dose of 0.6 mg kg^−1^ (Figure 6a). Next, we tested the antitumor efficacy of SA‐E‐MMAE against RG2 xenografts in nude mice. For this experiment, we further increased the SA‐E‐MMAE dose to 1 mg kg^−1^. Similar to 4T1 results, we observed a significant reduction in the tumor volume, around 80%, compared to the control (Figure 6c). While a slight weight loss was observed at the beginning of the therapy, overall, the mice tolerated 5 doses of SA‐E‐MMAE injections at 1 mg kg^−1^ (Figure 6d). Finally, we tested the SA‐E‐MMAE in HCT‐116 tumor bearing mice. We again applied 5 doses (0.8 mg kg^−1^ of MMAE), but this time we started the treatment when the tumor volume was around ≈50–100 mm^3^ on day 11 to investigate if SA‐E‐MMAE treatment can result in tumor shrinkage. All mice that received SA‐E‐MMAE showed a reduction in the tumor size (Figure 6e), which significantly improved the overall survival (Figure 6f). Average tumor volume was ≈150 mm^3^ at the end of SA‐E‐MMAE treatment on day 21, which gradually decreased to ≈40 mm^3^ on day 33 and remained almost unchanged for 2 weeks. While tumors in 3 of the mice started to grow again on days between ≈50 and 80, one mouse remained tumor‐free on day 120. Overall, these results suggest that SA‐E can be conjugated to chemotherapy drugs to improve their therapeutic index and antitumor activity.

## 3 Conclusion

In summary, we present a new mechanism for weakly assembled PA nanostructures (SA‐E) to specifically accumulate in a broad range of solid tumors based on their in situ assembly with plasma biomolecules and cell membranes. The proposed cancer targeting mechanism of SA‐E is summarized in Figure 4. In circulation, SA‐E disassembles and reassemblies mainly with LPs and to a lesser extent with albumin (Figure 1c–h). Hitchhiking on plasma components provides prolonged blood circulation (Figure 1i), allowing SA‐E to pass the tumor site numerous times and enabling their effective accumulation in the tumor microenvironment due to increased metabolism of solid tumors. In the tumor site, SA‐E internalizes into cancer cells mainly through binding to cell membranes, especially to cholesterol‐rich domains (Figure 2). Using these endogenous interactions, SA‐E demonstrated high tumor accumulation and retention in various mouse and rat models, including transgenic models, patient‐derived xenografts, orthotopic glioma tumors, and early lesions. In addition, we showed that SA‐E can improve the efficacy of a chemotherapy drug with reduced side effects. The findings of this study underscore the importance of interactions of self‐assembled nanomaterials with biomolecules on their fate in vivo and represent a novel way of exploiting these interactions to develop platforms with universal cancer‐targeting ability.

## 4 Experimental Section

### Materials

Peptides were commercially obtained from Genscript with purity >95%. The following reagents were used: ICG maleimide (Iris Biotech, RL‐2820), ICG (Fisher Scientific, I0535100MG), Cy5 maleimide (BroadPharm, BP‐22552), maleimide (Sigma‐Aldrich, 129 585), vcMMAE (MedChem Express, HY‐15575), MMAE (MedChem Express, HY‐15162), HDL from human plasma (Sigma‐Aldrich, SAE0054), LDL from human plasma (Sigma‐Aldrich, SAE0053), VLDL from human plasma (Millipore Sigma, 437 647), human serum albumin (Fisher Scientific, OSRHSA10), pooled human plasma (Innovative Research, IPLAWBK2E50ML), CellBrite Steady 488 (Biotium, 30106‐T), FITC‐dextran 70 (Santa Cruz Biotechnology, sc‐263323), brefeldin A (Alfa Aesar, J62340), bromsulphalein (MedChem Express, HY‐D0217), chlorpromazine hydrochloride (Sigma‐Aldrich, C8138), cis‐ACBD (Tochris, 0271), filipin complex (Sigma‐Aldrich, F9765), heparin sodium salt (Sigma‐Aldrich, H3149‐10KU), methyl‐β‐cyclodextrin (Sigma‐Aldrich, C4555‐5G), wortmannin (Alfa Aesar, J63983), bovine serum albumin (VWR, 0332), 4′,6‐diamidino‐2‐phenylindole (DAPI) (Sigma‐Aldrich, D9542), Hoechst stain (Invitrogen, H3570), 3‐(4,5‐dimethylthiazol‐2‐yl)‐2,5‐diphenyltetrazolium bromide (MTS) cell proliferation assay (Abcam, ab197010). Gadodiamide (GdDTPA‐BMA, Omniscan, GE Healthcare Inc. Marlborough, MA) MRI contrast was purchased through the OHSU in‐patient pharmacy. Manufacturers and catalog numbers of additional materials, such as antibodies, are given in the text below.

### Preparation of PA Dye or Drug Conjugates

Quantities of 4 mg of SA‐E (2.92 µmol) and 3.21 µmol (1.1 eq) of maleimide‐functionalized dyes, maleimide, or vcMMAE were mixed in 2 mL of anhydrous DMSO (Sigma Aldrich, 900645). The mixture was incubated at room temperature overnight under gentle shaking (300 rpm). The same protocol was used for conjugation reactions with SA‐Eb, SA‐K or No‐SA. The progression of the reaction was tracked using LC‐MS, employing an Acquity UPLC system (Waters) equipped with a SQ Detector 2 (Waters) and a reverse‐phase column (Waters, ACQUITY UPLC BEH C18 Column, 130 Å, 1.7 µm, 2.1 mm × 50 mm). A solvent gradient from water/acetonitrile (95:5) with 0.1% formic acid to water/acetonitrile (5:95) was run over 9 min at a rate of 0.4 mL min^−1^. Then, purification of the reaction mixtures was conducted using HPLC (Waters 1525, binary HPLC pump) with a reverse‐phase column (Waters, XBridge BEH C18 OBD Prep Column, 130Å, 5 µm, 19 mm × 150 mm). The solvent gradient in the HPLC method was gradually adjusted from water/acetonitrile (95:5) to (5:95) with 0.1% trifluoroacetic acid over a 45 min run at a flow rate of 0.8 mL min^−1^. A UV/Visible detector (Waters 2489) was applied to monitor the absorbance of eluents at 230 nm. The solutions collected from the HPLC runs were concentrated and lyophilized. Finally, purified conjugates were dissolved in sterile PBS (10 mm, pH 7.4) at concentrations between 1 and 2.5 mm and stored at −20 °C.

### TEM Imaging of PA Nanostructures

For TEM imaging, PAs conjugated with ICG, Cy5 or MMAE were dissolved in ultrapure water at a concentration of 200 µm. Carbonfilm 200 copper mesh TEM grids were glow‐discharged first. Then, PA solutions (15 µL) were placed on the grids, incubated for 10 min before blotting, and air‐dried. 15 µL of ultrapure water was applied to wash the grid for 3 min. After incubation, the water was removed by blotting from the edge of the grid. For negative staining of the samples, a 0.4% uranyl acetate solution was prepared in ultrapure water and filtered using a 0.1 µm syringe filter. 15 µL of filtered uranyl acetate solution was applied to the grid and incubated for 5 min. Finally, TEM grids were blotted to remove excess stain and dried at room temperature. TEM imaging was performed using a Tecnai or Talos microscope (FEI).

### Fluorescence Polarization of PA Nanostructures

ICG (10 mol%) containing PA micelles were prepared by mixing ICG‐conjugated PAs with unconjugated or MMAE‐conjugated PAs to achieve a final concentration of 20 µm ICG‐conjugated PAs and 200 µm of total PAs. Aliquots of 100 µL were added to a 96‐well plate (3 wells for each PA). Fluorescence polarization measurements were performed using a microplate reader (TECAN Spark 20 M). The corrected parallel intensity (*I*_par_) and perpendicular intensity (I_per_) were used to calculate the fluorescence anisotropy (r) using the following equation: *r* = (*I*_par_–*I*_per_)/(*I*_par_ + *I*_per_).

### Stability of PA Nanostructures in SDS Solution

ICG conjugated PAs were dissolved in PBS and diluted into either water or 5% SDS to final concentrations of 100 µm. 100 µL samples were added to a 96‐well plate (3 wells for each PA). Fluorescence spectra of ICG were recorded separately using a microplate reader (TECAN Spark 20 m).

### Stability of PA Nanostructures in Plasma

ICG or Cy5 conjugated PAs were dissolved in PBS (10 mm, pH 7.4) at a concentration of 1 or 100 µm, and 90 µL aliquots of these solutions were added to a 96 well plate (4 wells for each PA). Then, 10 µL of pooled human plasma was added to each well, and fluorescence spectra of ICG or Cy5 were recorded using a microplate reader (TECAN Spark 20 M) at different time points.

### FPLC Experiments

FPLC experiments were performed using SA‐E, SA‐K, or No‐SA conjugated with ICG or SA‐E conjugated with MMAE at a final concentration of 250 µm in filtered PBS (10 mm, pH 7.4) with or without human plasma or purified LPs or albumin. For analysis of SA‐E interactions with purified LPs, VLDL, LDL, HDL, and human serum albumin were diluted in filtered PBS to approximate concentrations found in 10% human plasma: 0.0025, 0.13, 0.05, and 5mg mL^−1^, respectively. SA‐E was incubated with the purified LP/protein mixture for 30 min at 37 °C before injection. For analysis of plasma interactions, PAs or No‐SA were incubated in PBS containing 10% pooled human plasma for 30 min at 37 °C before injection. The total volume injected for all samples was 400 µL. All data was collected on an ÄKTA pure chromatography system using the following running conditions: size exclusion chromatography on a Superose 6 Increase 10/300 GL column with a column volume (CV) of 30 mL, pre‐column pressure limit of 5 MPa, delta‐column pressure limit of 2.6 MPa, flow rate of 0.75 mL min^−1^, in 100% PBS. The sample running method included first an equilibration step before injection of the sample with 100% PBS for 0.5 CV, followed by sample application via 1 mL capillary loop in 100% PBS, and finished with a linear elution consisting of 2 CV collected in 1 mL fractions. The UV‐Vis detector wavelengths were 250, 280, and 700 nm to detect MMAE, all proteins/LPs, and ICG, respectively.

### Mass Spectrometry

Samples were prepared for mass spectrometry analysis via a pull‐down of a biotinylated version of the SA‐E probe (SA‐Eb, 100 µm) incubated with 50% pooled human plasma at 37 °C for 2 h. Before incubation with the SA‐Eb‐plasma mixture, magnetic streptavidin‐coated beads (Dynabeads MyOne Streptavidin T1, Invitrogen, 65601) were washed with PBS (10 mm, pH 7.4) then blocked with 1% casein equal to the total initial volume of beads for 15 min at room temperature. After blocking, beads were washed again with PBS. Beads were then combined with SA‐Eb‐plasma solution, 25 µL beads to 200 µL mixture. This mixture was then incubated at room temperature for 30 min. After incubation, the remaining solution was removed, and the beads were washed five times with PBS. Bound SA‐Eb was eluted from beads in 30 µL 0.5% Triton X‐100 solution. Magnetic beads were then removed, and the elution was collected for analysis. For plasma control, the incubation with SA‐Eb step was skipped, and 50% plasma in PBS was incubated with beads and washed and eluted using the same method as the SA‐Eb‐plasma solution. The elution solutions were then analyzed by the Proteomics Shared Resource facility at Oregon Health & Science University (See Supporting Information for details).

### Cell Lines

Cell lines used for the experiments were all tested for Mycoplasma at least once every other year. The following cell lines were used: HCT‐116 (human colorectal cancer, ATCC CCL‐247), A375‐Luc/iRFP (human melanoma, Creative Biogene CSC‐RR0254), MCF7 (human breast cancer, ATCC HTB‐22), 4T1 (mouse breast cancer, ATCC CRL‐2539), RG2 (rat glioma, ATCC CRL‐2433), and LL/2 (mouse lung cancer (LLC1), ATCC CRL‐1642). MC38 mouse intestinal epithelial cancer cells were provided by Dr. Melissa Wong at OHSU. SR‐B1‐/‐ and wild type TRAMP‐C2 mouse prostate cancer cells were provided by Jonathan D. Smith at Case Western Reserve University. LDL‐R‐/‐ and wild type HeLa human cervical cancer cells were purchased from Ubigene (YKO‐H1027). Patient derived pancreatic or colon cancer cells were obtained at OHSU with patient consent and cultured for at least 5 passages. Cell lines were maintained in either RPMI Medium 1640 (Gibco, 11875093) supplemented with 10% fetal bovine serum (FBS) (Cytiva, SH30396‐03) and 5% penicillin‐streptomycin (pen‐strep) (Gibco, 15140163) or DMEM with 4.5 g/L D‐glucose, L‐glutamine, and 110 mg/L sodium pyruvate (Gibco, 11995065) and supplemented with 10% FBS and 5% pen‐strep as the growth media in the incubator at 37 °C under 5% CO_2_. For mCherry transfection of RG2 cells, lentivirus targeting mCherry to the cell membrane (Takara, 0026VCT, rLV.EF1.mCherry‐Mem‐9) was used according to manufacturer's recommendation. Transfected cells were passaged at least 10 times before being used for in vivo experiments.

### Cellular Uptake Studies

To compare the uptake of SA‐E and SA‐K, 1 × 10^5^ 4T1 cells were grown to near confluency (1–2 days) in 96 well plates with black walls and flat glass bottom. 100% Cy5 conjugated PAs (20 or 10 µm) or 10% Cy5 conjugated PA micelles (10 µm Cy5‐PAs mixed with 90 µm maleimide blocked labeled PAs) were preincubated in 10% FBS containing RPMI for 30 min at 37 °C, wells were aspirated, and 100 µL of media containing PAs were added. The plate was incubated for 30 min for the experiments with 100% Cy5 conjugated PAs. Media were aspirated, wells were washed 3x with 150 µL sterile PBS, and then 100 µL RPMI containing 10% FBS was added to each well. The plate was incubated for 1 h at 37 °C. For 10% and 100% Cy5 micelle uptake comparison, plate was incubated for 2 h at 37 °C with PAs. Then, media containing PAs were aspirated and washed with PBS, as above. Cell nuclei were stained with Hoechst (1:5000 dilution in PBS) for 5 min and then removed. 100 µL of phenol red free RPMI containing 10% FBS was added to each well. Confocal images were obtained with a Nikon CrestOptics X‐Light V3 Spinning Disk Confocal microscope under 20x and 60x objective using NIS‐Elements software (405 nm laser for Hoechst and 637 nm laser for Cy5 imaging). The microscope incubator was preheated for 30 min to reach 35 °C prior to imaging.

To study the uptake of SA‐E or SA‐K micelles (10% or 100% Cy5 micelles) or No‐SA over time, 4T1 cells were grown to near confluency in a 96 well plate as described above. Cy5 conjugated 100% Cy5 conjugated PAs (20 µm), 10% Cy5 conjugated PAs (10 µm Cy5), or No‐SA (20 µm) or were preincubated in 10% FBS containing RPMI for 30 min at 37 °C. Cell nuclei were stained with Hoechst stain as above. Cell membranes were stained (CellBrite 488, 1:1000 in 10% FBS containing RPMI) for 30 min at 37 °C. Wells were aspirated, and 100 µL of PAs were added to the wells for ≈1 min. Wells were washed with sterile PBS (3x, 150 µL), and Confocal images were taken at different time points (up to 2 h) after PA or No‐SA addition as described above using a 60x objective.

To study the endocytosis of Cy5 conjugated SA‐E, 4T1 cells were grown to near confluency in a 96 well plate as described above. FITC‐dextran (2 mg mL^−1^) and Cy5 conjugated SA‐E (20 µm) were preincubated in 10% FBS containing RPMI for 30 min at 37 °C. Wells were aspirated, and 100 µL of media containing FITC‐dextran and Cy5 conjugated SA‐E was added. The plate was incubated at 37 °C for 4 h. Wells were washed with PBS, nuclei were stained with Hoechst, and Confocal imaging was performed as described above. A 477 nm laser was used for FITC imaging.

To study the effect of serum biomolecules on cellular uptake of SA‐E, 4T1 cells were grown to near confluency in a 96 well plate as described above. Cy5 conjugated SA‐E (20 µm) was preincubated in RPMI with or without 10% FBS for 30 min. Wells were aspirated, and 100 µL of media containing Cy5 conjugated SA‐E was added. Then, the plate was incubated with SA‐E for 30 min and washed with PBS as described above. 100 µL of cell media, either with or without FBS as appropriate, was added to each well, and the plate was returned to the incubator for 1 h. To stain cell nuclei, a 1:5000 dilution of Hoechst stain was made in serum free media and added to each well. The plate was incubated for 5 min and fluorescence images were taken using a Leica Thunder DMi8 fluorescent microscope using LAS X software.

For SR‐B1 studies, an equal number of wells in a black walled, glass bottom 96‐well plate were seeded with either wild type or SR‐B1‐/‐ TRAMP‐C2 cells and grown to near confluency, as above. The Cy5 conjugated SA‐E was diluted in 10% FBS containing DMEM and incubated at room temperature for 30 min. All media from the wells was aspirated. Four wells of each cell type received 100 µL aliquots with SA‐E (20 µm). The plate was incubated for 30 min, as above. Following the methods above, wells were washed with PBS, media was added, and the plate was returned to the incubator for 1 h. Finally, Hoechst staining and fluorescence imaging were performed using a Leica Thunder DMi8 fluorescent microscope, as described above.

For LDL‐R studies, 2 × 10^5^ wild type or LDL‐R‐/‐ HeLa human cervical cancer cells were grown to near confluency in 96 well plates with black walls and flat glass bottom. Cy5 conjugated SA‐E (20 µm) was preincubated in DMEM containing 10% FBS for 30 min at 37 °C. Wells were aspirated, and 100 µL of the SA‐E containing DMEM was added. The plate was incubated for 30 min, as above. The wells were washed with sterile PBS, as above, media was added, and the plate was returned to the incubator for 1 h. Wells were aspirated, and nuclei were stained with Hoechst as above. Wells were aspirated, and 100 µL of phenol red free DMEM with 10% FBS was added to each well. Cells were then imaged for Cy5 conjugated SA‐E with a Zeiss Apotome 3 microscope at 20x and 63x objectives using Zen software.

To explore the effect of serum proteins and different inhibitors on the cellular uptake of SA‐E, 4T1 cells were grown to near confluency in 96 well plates, as described above. For cellular uptake mechanism studies, the growth media (RPMI containing 10% FBS) was exchanged for 100 µL of FBS free media, then returned to the incubator. Inhibitors (brefeldin A, bromsulphalein, chlorpromazine hydrochloride, cis‐ACBD, filipin, heparin, methyl‐β‐cyclodextrin, or wortmannin) were made in FBS free media at a concentration of 1 mm. Then, 1 µL of media containing inhibitors was added to wells, so that the final drug concentration in each well was 10 µm. The plate was returned to the incubator for 20 min. After incubation, Cy5 conjugated SA‐E was added to all wells such that the final concentration was 20 µm per well. The plate was returned to the incubator for 30 min. Wells were aspirated and washed with PBS as above. 100 µL of FBS free media was added, and the plate was returned to the incubator for 1 h. Finally, Hoechst staining and fluorescence imaging were performed using a Leica Thunder DMi8 fluorescent microscope, as described above.

### Mouse Tumor Models

All experiments were approved by the Oregon Health and Science University (OHSU) Institutional Animal Care and Use Committee (IACUC) (TR01_IP00000674) and conformed to the guidelines set by the United States Animal Welfare Act and the National Institutes of Health. Mice were aged between 3 and 8 months and 20–30 g in weight and were housed in specific pathogen free cages. Human, mouse, or rat cells were injected into nude (The Jackson Laboratory, 002019), NSG mice (The Jackson Laboratory, 005557), Balb/c mice (The Jackson Laboratory, 000651), or C57Bl/6J mice (The Jackson Laboratory, 000664), as specified below. Cells were injected in 100 µL sterile DMEM at a concentration of 0.5–1×10^7^ cells mL^−1^, unless otherwise specified. For tumor accumulation and biodistribution studies, A375 cells were injected intradermally into the left ears of NSG mice (The Jackson Laboratory, 005557). MCF7 cells were injected into the mammary fat pad of NSG mice. HCT‐116, RG2, and HeLa (wild type or LDL‐R knockout) cells were subcutaneously injected into the left or both flanks of NSG mice. 4T1 cells were injected into the mammary fat pad of Balb/c mice. LLC1, MC38, and TRAMP‐C2 (wild type or SR‐B1 knockout) were injected into C57Bl/6J mice. Patient‐derived xenografts were generated by injecting the patient‐derived cancer cells subcutaneously into the left flank of NSG mice. ApoA1 and LDL‐R knockout mice were purchased from the Jackson Laboratory (002055 and 002207, respectively). MC38 cells were subcutaneously injected into both flanks of these mice. Apc^min^ and MMTV‐PyMT mice were purchased from the Jackson Laboratory (002020 and 002374). For antitumor activity studies, 4T1 cells were injected into the mammary fat pad of Balb/c mice. HCT‐116 and RG2 cells were subcutaneously injected into the left flanks of nude mice. For antitumor activity studies, HCT‐116 cells were injected in a 1:1 mixture of DMEM and Matrigel (Life Science 354263).

### Blood Circulation in Mice

For blood circulation experiments, Balb/c mice were injected with 100 µL of ICG conjugated PAs or No‐SA, or free ICG (0.5 mm). Then, blood was drawn via the saphenous vein or retro‐orbitally at different time points (immediately after injection to 1 day). 2 µL of blood was diluted into 98 µL of PBS (10 mm, pH 7.4), and ICG fluorescence was measured using a microplate reader (TECAN Spark 20 M) at excitation and emission wavelengths of 745 and 820 nm, respectively. Blood from wild type mice was collected to prepare calibration curves for each probe. To each 18 µL aliquot of blood, 2 µL of probe stock solution (0.5 mM) was added and incubated at 37  °C for 1 h. The spiked samples were then serially diluted in blood between 50 and 0.1 µm. For fluorescence measurement, 2 µL of each diluted sample was further diluted into 98 µL of PBS, and ICG fluorescence was measured using a TECAN plate reader.

### Collection of Blood Components for FPLC and Fluorescence Measurements

For these experiments, 100 µL of ICG conjugated SA‐E (0.5 mm) was intravenously injected into wild type (C57Bl/6J), ApoA1 ‐/‐, or LDL‐R ‐/‐ mice. One hour after injection blood was collected via cardiac puncture (500 µL) in EDTA containing tubes (BD MicroTainer Blood Collection Tube, 365974). To separate plasma, blood samples were centrifuged for 10 min at 1500 g, and supernatants were removed. Samples were centrifuged again for 10 min at 2500 g, and supernatants were stored at −20 °C for up to 5 days before use. For RBCs, 10 µL of the centrifugation pellet was separated and stored at −20 °C for up to 5 days before analysis. For WBCs, 100 µL of the remaining pellet was collected and combined with 2 mL Red Blood Cell Lysis Buffer (BioLegend, 420514). This mixture was allowed to incubate at room temperature for 15 min with occasional mixing. The solution was then centrifuged at 500 g for 5 min, the supernatant was removed, and the pellet was redissolved in 30 µL PBS, then stored at −20 °C for up to 5 days. FPLC of plasma samples was performed as described above using an ÄKTA pure chromatography system. For fluorescence measurements, 5 µL of samples were added to a 96 well plate, diluted to a total volume of 100 µL with PBS, and fluorescence was measured using a microplate reader (Tecan Spark 20 M).

### Tumor Accumulation in Mice

For tumor accumulation studies in subcutaneous (HCT‐116, A375, TRAMP‐C2, MC38, LLC1, RG2, PDX cell lines, wild type or SR‐B1 ‐/‐ TRAMP‐C2, and wild type or LDL‐R ‐/‐ HeLa), intradermal (A375), or fat pad (4T1 and MCF‐7) tumors, cells were implanted as described above. When the tumors reached a size around ≈0.3–1 cm, mice were injected intravenously with 100 µL of ICG conjugated PAs (in PBS, 0.5 mm) or free ICG (in PBS, 0.5 mm). After probe injections, mice were imaged using the IVIS Spectrum (PerkinElmer) with excitation and emission wavelengths of 745 and 820 nm, respectively, at the specified time points. Living Image (PerkinElmer) software was used for all analyses. For MMTV mice experiments, mice were injected intravenously weekly with SA‐E (100 µL PBS, 0.5 mm) between 71 and 85 days of age and imaged 2 days later in the IVIS. For Apc^min^ mice experiments, mice were aged to 4 months to allow intestinal tumors to form. SA‐E was injected intravenously (100 µL PBS, 0.5 mm), 2 days later the intestinal tissue was harvested, washed, and analyzed in the IVIS for ICG.

### PA Fluorescence in Liver Homogenates

Liver samples (6.2 g total weight) collected from healthy BALB/cJ mice were placed in a gentleMACS M Tube containing 2 mL of PBS. The M Tube was fitted with a 600 µm mesh strainer, and homogenization was performed using a gentleMACS Dissociator by running a one‐minute program at 1260 rotations per round (rpr) for five cycles. ICG conjugated SA‐E and SA‐K were then spiked into the liver homogenates in a 96‐well plate at final concentrations of 0, 0.1, 0.5, 2.0, 5.0, and 10 µm. Fluorescence signals from the homogenates were measured using an IVIS (PerkinElmer).

### Biodistribution in Mice

For biodistribution studies in 4T1 and MC38 tumor models in wild type, ApoA1 ‐/‐, or LDL‐R ‐/‐ mice, 100 µL of ICG conjugated PAs or free ICG (0.5 mm) were intravenously injected. 2 days after injection, the mice were euthanized, and tumors and organs were excised and analyzed in the IVIS (PerkinElmer) for ICG.

To determine the percentage of the injected dose of SA‐E in tissues, 4T1 tumor, spleen, kidney, lung, and colon tissues were harvested from 5 mice 2 days after ICG conjugated SA‐E (IV, 100 µL, 0.5 mm). Organ weights were measured for normalization. Tissues were homogenized in gentleMACS M Tube containing 1 mL of PBS and equipped with a 600 µm mesh strainer using a gentleMACS dissociator as described above. The resulting tissue homogenates were transferred into a 96‐well plate for fluorescence measurement using a microplate reader (TECAN Spark 20 m). Additionally, organ tissues from healthy mice without probe injection were harvested and homogenized to prepare calibration curves. ICG conjugated SA‐E was spiked into tissue samples at different concentrations, and fluorescence intensities were measured using a microplate reader (TECAN Spark 20 M). For biodistribution studies with 10 mol% ICG containing micelles, ICG conjugated PAs were mixed with maleimide blocked PAs to achieve a final concentration of 0.25 mm ICG conjugated PAs and 2.5 mm total peptides. Their tumor accumulation and biodistribution were studied as described above.

### Clearence Study

Six Balb/c mice were intravenously injected with 100 µL of either ICG‐conjugated SA‐E or PBS, with three mice in each group. Urine and stool samples were collected from each mouse at different time points up to 7 days. Urine samples were diluted 10‐fold in PBS, and ICG fluorescence was measured using a microplate reader (TECAN Spark 20 M). Urine from healthy Balb/c mice was collected and pooled to generate a calibration curve. Fluorescence of stool samples was detected using an IVIS (PerkinElmer).

### Toxicity in Mice

Wild type mice were injected intravenously with ICG conjugated SA‐E at 4 times the dose used previously for imaging (100 µL, 2 mm). 1 day later, blood was removed via cardiac puncture, placed into an EDTA tube, and then centrifuged at 1500 g for 10 min, then 2500 g for 10 min to obtain plasma. The plasma was analyzed for Aspartate Aminotransferase activity (AST) (Abcam, AB105135) and creatinine (Crystal Chem, 80350) following the manufacturer's protocol. For complete blood counts, whole blood (10 µL) was mixed with 2 mm EDTA (10 µL) and analyzed using the HemaVet 950S (Drew Scientific).

### Flow Cytometry

RG2‐mCherry cells (100 µL sterile DMEM, 0.5 × 10^7^ cells mL^−1^) were implanted subcutaneously in nude mice and allowed to grow to 0.5–1 cm. Mice were then injected intravenously with the Cy5 labeled SA‐E (100 µL, 0.5 mm). 2 days later, tumors were extracted, minced, and dissociated using 1 mg mL^−1^ solution collagenase/dispase (Roche, 10269638001 and 11097113001) for 45 min at 37 °C. The solution was broken down with pipetting and filtered through a 70 µm filter. The single‐cell solution was suspended in sterile PBS (10 mm, pH 7.4) and then incubated with 1:50 dilution of AlexaFluor 488 Rat Anti‐Mouse CD45 antibody (BD Pharminogen, 567377) for 45 min at room temperature. Finally, the cells were pelleted and, washed and incubated with 5 µM Calcein Blue, AM (Invitrogen, C1429) for 20 min. The cells were then analyzed in BD FACSymphony in the DAPI channel (355 nm laser, 450/50 filter), FITC channel (488 nm laser, 515/30 filter), Cy3 channel (561 nm laser, 586/15 filter), and Cy5 channel (628 nm laser, 670/30 filter). Cells in culture were used to establish size and mCherry intensity. Tumors from mice that did not receive any SA‐E injection and unstained cells were used as controls for the gating of SA‐E and CD45. Finally, three experimental mice were analyzed in all channels using the gating established from the controls. Gating was performed in FlowJo software.

### Rat Models

The care and use of animals were approved by the Institutional Animal Care and Use Committee and were supervised by the OHSU Department of Comparative Medicine (DCM) (protocol IP00000843). Female Long Evans rats (200–300 g) were purchased from Charles River Laboratories by OHSU DCM. Rats were anesthetized using 3% isoflurane, and a 2 mm diameter hole was drilled in the skull using a 27‐gauge needle in a stereotactic frame (David Kopf Instruments; Tujunga, CA). Cancer cells (10^6^ cells/10 µL, >90% viability) were injected at a rate of 1 µL min^−1^. Rat RG2 cells with or without mCherry were implanted into the right caudate nucleus (bregma = 0; lateral 0.31 cm; vertical – 0.65 cm).

### MRI of Rat Tumors

MRI was performed to confirm tumor growth prior to administration of SA‐E using a horizontal bore 11.75 T magnet (Bruker Scientific Instruments, Billerica MA) maintained by OHSU Advanced Imaging Research Center (AIRC). Animals were imaged 8–15 days after tumor implantation. Under intraperitoneal dexmedetomidine (Zoetis, 0.5 mg kg^−1^) and ketamine (Zoetis, 60 mg kg^−1^) sedation, the lateral caudal vein was catheterized, and the animal was securely placed in a Plexiglas holder and positioned in the small animal MRI. T1 (Fat saturated 2D Spine Echo [SE], TR 160, TE 1.654), T2 (2D SE, TR 4020.62, TE 23.64) and T2* (2D Gradient Echo, TR 430.25, TE 6.656, FA 30) sequences were obtained before a dose of 50 µmol kg^−1^ of Omniscan via tail vein catheter followed by a ≈0.25 mL saline flush. A post‐contrast T1 scan was repeated 5 min following the administration of Omniscan.

### IVIS and Confocal Imaging of Rat Tumors

After confirmation of RG2 tumor growth, ICG conjugated SA‐E (1 mL, 0.5 mm) was delivered via tail vein catheter, followed by a ≈0.25 mL saline flush, before anesthetic reversal with intraperitoneal atipamezole HCl (Antisedan, 1.0 mg kg^−1^). For IVIS imaging of rat tumors, animals were euthanized with euthasol (Virbac) via intracardiac injection under isoflurane anesthetization 2 days after SA‐E administration. Brain, lung, liver, spleen, and kidney were harvested and stored in chilled PBS until IVIS imaging (≈30 min after resection). Tissues were first imaged in the IVIS for the ICG signal. Then, wholemount brain sections were imaged under IVIS for the ICG signal of SA‐E and the mCherry signal of RG2 cells. For Confocal imaging of brain sections, the brain was bisected through the tumor, flash frozen, and stored at −80 °C in O.C.T. embedding medium (Sakura Finetek USA Inc, 4583). The tissue was then sectioned with a cryostat (Leica CM 3050s) at 15 µm thickness and stored on dried ice before fixing in 4% formaldehyde for 5 min. Tissue sections were rinsed in PBS and then counterstained with Hoechst stain (1:4000 dilution in PBS) for 5 min and imaged on a Nikon CrestOptics X‐Light V3 Spinning Disk Confocal microscope (Hoechst 405 nm laser, mCherry 545 nm laser, ICG 748 nm laser) with 20x and 60x objective using NIS‐Elements software.

### Cytotoxicity of SA‐E‐MMAE

To assess the cytotoxicity of SA‐E‐MMAE and free MMAE, 4T1 cells, and RG2 cells were seeded in 96‐well plates at a density of 5 × 10^3^ cells per well in 100 µL of RPMI (10% FBS). The plates were then incubated at 37 °C under 5% CO_2_ for 1 day before the addition of SA‐E‐MMAE or free MMAE, resulting in final drug concentrations ranging from 0 to 10 µm for SA‐E‐MMAE and 0 to 0.05 µm for MMAE. Plates were further incubated at 37 °C under 5% CO_2_ for 3 days. To evaluate cellular viabilities, the RPMI media in each well was replaced with fresh RPMI containing 10% MTS reagent. Subsequently, plates were incubated at 37 °C for 2 h before measuring the MTS absorbance at 490 nm using a microplate reader (TECAN Spark 20 m).

### Antitumor Effect of SA‐E‐MMAE

Mice were injected with 4T1, RG2, or HCT‐116 cells as described above. Mice were then treated intravenously with the free MMAE (0.1 or 0.3 mg kg^−1^), SA‐E‐MMAE (0.3, 0.6, 0.8, or 1 mg kg^−1^) or PBS (10 mm, pH 7.4). 5 doses were given every 2–3 days over 11 days. For 0.3 mg kg^−1^ dose of free MMAE, the drug was administered 3 times as a significant weight loss was observed after the third injection. Tumor size and mouse weight were monitored 2–3 times per week until a humane endpoint (tumor size ≈2 cm or >20% body weight loss) or the study endpoint (18, 25, or 120 days for 4T1, RG2, and HCT‐116 studies, respectively). Tumor size was measured in two directions with a caliper rounded to the nearest tenth of a mm. Volume was calculated as a basic sphere of 4/3πr^3^.

### Statistical Analysis

Statistical analysis was performed using OriginPro 20024 (OriginLab). Data are presented as mean ± standard error of the mean (SEM). Studies were run in at least triplicates, unless otherwise stated in the figure captions. Statistical analysis was performed using one‐way analysis of variance (ANOVA), Student's *t*‐test, or log‐rank analysis. Statistical analysis method used and sample size (n) for each experiment were specified in figure captions. Statistical significance was set at ^*^ *p* < 0.05, ^**^ *p* < 0.01, and ^***^ *p* < 0.001.

## Conflict of Interest

A.Y., J.M.F., and B.P.B submitted a patent application (PCT/US2022/07 3964) on the early findings of this study. The other authors declare no competing interests.

## Supporting information



Supporting Information

Supplemental Video 1

## Data Availability

The data that support the findings of this study are available from the corresponding author upon reasonable request.
